# Sample environment for *operando* solid-state battery characterization

**DOI:** 10.1107/S1600576726000853

**Published:** 2026-03-20

**Authors:** Therese S. S. Faurskov, Lasse N. Skov, Jakob B. Grinderslev, Lasse R. Kristensen, Jens Magnus Horsted Bendtsen, Mads Kofod Dahl, Tommy Kessler, Bettina Pilgaard Andersen, Innokenty Kantor, Mads R. V. Jørgensen, Dorthe B. Ravnsbæk, Torben R. Jensen

**Affiliations:** a Interdisciplinary Nanoscience Center (iNANO) and Department of Chemistry Aarhus University Langelandsgade 140 Aarhus C 8000 Denmark; b Department of Electrical and Computer Engineering Aarhus University Finlandsgade 22 Aarhus N 8200 Denmark; c MAX IV Laboratory Lund University Fotongatan 2 Lund 224 84 Sweden; d Department of Physics Technical University of Denmark Fysikvej 3 Kgs. Lyngby 2880 Denmark; HPSTAR and Harbin Institute of Technology, People’s Republic of China

**Keywords:** solid-state batteries, powder diffraction, *operando*, *in situ*, X-ray scattering

## Abstract

All-solid-state batteries provide new hope for developing electrical energy storage far beyond the current state of the art. Here, we present a new sample environment developed for *operando* and *in situ* investigations of solid-state batteries, allowing for simultaneous collection of X-ray diffraction and electrochemical data along with temperature control and stack pressure monitoring. The Aarhus pressure temperature *operando*X-ray, APTOX, cell has a 35° 2θ opening, allowing for measurement of high-quality powder X-ray diffraction data to obtain valuable information on structural changes during battery cycling. We present two different variants of the APTOX cell: APTOX-Pmon with the ability to monitor the applied stack pressure for advanced applications and APTOX-Spring with a spring system to apply constant mechanical pressure. The APTOX cell is compatible with different X-ray window configurations, allowing for tailoring of the X-ray background and attenuation, as well as different physical properties depending on the optimal conditions for the system of interest. Finally, we demonstrate the applicability by presenting *operando* powder X-ray diffraction data obtained using both an in-house diffractometer equipped with an Ag *K*α_1_X-ray source and a synchrotron source at MAX IV, Lund, Sweden.

## Abstract 2

A versatile sample environment enabling *operando* studies of solid-state batteries with temperature control and pressure monitoring is described.

## 1. Introduction

Batteries are increasingly indispensable in modern applications such as electric vehicles, portable electronic devices and energy storage. In recent years, solid-state batteries (SSBs) have attracted a lot of attention, due to their potential for more compact and safer energy storage when compared with today’s Li-ion technology. This has led to the development of several new solid electrolytes, all with different thermal and electrochemical operating windows (Liu *et al.*, 2023 ▸; Hou *et al.*, 2020 ▸; Zhan *et al.*, 2023 ▸; Manthiram *et al.*, 2017 ▸; Jiang *et al.*, 2023 ▸; Tao *et al.*, 2023 ▸; Nikodimos *et al.*, 2023 ▸; Ren *et al.*, 2023 ▸; Brighi *et al.*, 2020 ▸; Pang *et al.*, 2022 ▸). SSBs are expected to provide multiple advantages over state-of-the-art Li-ion batteries. The solid electrolyte in SSBs allows for compact bipolar stacking and potential use of metal anodes, which is expected to provide high power and energy densities (Kato *et al.*, 2016 ▸; Janek & Zeier, 2023 ▸). Furthermore, improvements to safety and lifetime are expected due to the removal of the flammable and unstable liquid organic electrolyte. A solid inorganic electrolyte may also operate in a larger temperature range than organic liquid electrolytes and may allow the SSB to be operated with faster charging and discharging rates (Kato *et al.*, 2016 ▸). Research on SSBs is in addition exploring an emerging class of solid electrolytes which offer high ionic conductivity and stability against alkali metal anodes, namely metal *closo*-hydridoborates. These have been demonstrated in SSBs such as Na|Na_4_(B_12_H_12_)(B_10_H_10_)|NaCrO_2_ (Duchêne *et al.*, 2017 ▸) and InLi|Li_3_(CB_11_H_12_)_2_(CB_9_H_10_)|NMC811 (Braun *et al.*, 2024 ▸). Furthermore, researchers are studying new chemistries based on more abundant elements and with divalent charge carriers such as Mg^2+^, *e.g.* the recently demonstrated all-solid-state magnesium batteries using a Mg-metal anode, a TiS_2_ cathode, and composites of Mg(BH_4_)_2_·1.6NH_3_ or Mg(BH_4_)_2_·1.5THF with MgO nanoparticles as the solid electrolyte (Skov *et al.*, 2022 ▸; Skov *et al.*, 2023 ▸). A challenge for SSBs is to maintain good contact at the solid interfaces between the electrolyte and electrodes during charge and discharge as chemical reactions may introduce volume changes. Therefore, an external mechanical pressure is often applied to SSB cells to increase the contact between solid layers and the stability of the electrochemical cell (Zhang *et al.*, 2017 ▸).

This interest in SSBs has prompted an increased demand for advanced, non-destructive characterization of battery cells during charge and discharge. X-ray techniques provide powerful methods (diffraction, imaging and spectroscopy) for detailed investigation of the multiscale complexity, chemical reactions and interphase formation that may occur in all-solid-state batteries (Borkiewicz *et al.*, 2015 ▸). Early studies mainly used *ex situ*/*post mortem* techniques, where samples were investigated before and after a chemical reaction, *i.e.* after charge or discharge, by disassembly of the battery cell. However, these techniques provide no insight into the dynamic reaction mechanisms. Investigation of chemical reactions *in situ* and under dynamic conditions, such as variable temperatures, gas pressure and composition, or under hydro­thermal conditions have been successfully conducted over the past two decades (Jensen *et al.*, 2010 ▸; Hansen *et al.*, 2015 ▸; Møller *et al.*, 2014 ▸). In recent years, *operando*/*in situ* characterization methods for batteries have also been developed to investigate devices under working conditions. Today, *operando* investigations of batteries involve measuring the diffracted X-ray beams and a variety of other parameters to link structural and electrochemical properties (Borkiewicz *et al.*, 2015 ▸; Golozar *et al.*, 2021 ▸). An assortment of cells have been constructed to facilitate *operando*X-ray diffraction investigations for battery research (Llewellyn *et al.*, 2020 ▸). The AMPIX cell is a widely used electrochemical cell for *operando*X-ray scattering and absorption spectroscopy synchrotron experiments (Borkiewicz *et al.*, 2012 ▸). Other approaches to obtain *operando* powder X-ray diffraction (PXRD) data for batteries with liquid electrolytes include a capillary-based setup and modified coin cells and pouch cells (Johnsen & Norby, 2013 ▸; Herklotz *et al.*, 2016 ▸; Graae *et al.*, 2023 ▸). Furthermore, the recent DANOISE cell, which is inspired by the AMPIX cell, has been demonstrated as an alternative for in-house *operando* PXRD and absorption spectroscopy studies (Johansen *et al.*, 2024 ▸). The Leriche cell is another *operando* cell used for PXRD investigations of batteries (Leriche *et al.*, 2010 ▸). It was originally demonstrated using a liquid electrolyte but has since been modified to improve the mechanical stability and electrical isolation, and it has been used to obtain in-house *operando* PXRD data of a solid-state sodium battery (Jakobsen *et al.*, 2022 ▸).

The RATIX cell is a tubular *operando* cell for spatially resolved X-ray scattering and X-ray absorption spectroscopy experiments, which allows for adjustments of the stack pressure. It can be used to map individual components or layers of an electrode in the battery stack. While the cell offers spatial resolution, it lacks temperature control (Liu *et al.*, 2016 ▸). The DRIX cell also uses a radial geometry and is optimized for X-ray pair distribution function studies as well as depth profiling along the stacking direction of the cell. Furthermore, a custom-designed heater allows the DRIX cell to operate at elevated temperatures (Diaz-Lopez *et al.*, 2020 ▸). Finally, the SNBL cell is a Swagelok-type *operando* cell made for quasi-simultaneous X-ray diffraction and X-ray absorption spectroscopy experiments as well as in-house *operando* PXRD experiments (Sottmann *et al.*, 2016 ▸). The applicability of the SNBL cell has been demonstrated for *operando* synchrotron radiation powder X-ray diffraction (SR-PXRD) characterization of a solid-state lithium battery using an in-house-built heater (Kharbachi *et al.*, 2020 ▸). However, the cell offers limited control of the stack pressure and pressure monitoring.

With the increasing interest in SSBs, a new *operando* cell design is required. This cell should accommodate various sample thicknesses, apply a controlled uniform mechanical stack pressure and provide the temperature needed for optimal cycling conditions. This has prompted the present work on a new *operando* cell design, denoted the APTOX cell, *i.e.* ‘Aarhus pressure temperature *operando*X-ray’ cell, which is inspired by the AMPIX cell design. The APTOX cell provides a versatile sample environment with new functionalities, allowing for heating of the cell and simultaneous monitoring of temperature and mechanical pressure, along with the collection of electrochemical data during battery operation while measuring the diffracted X-ray beams. Thus, the correlation between structural changes, variation in mechanical pressure and electrochemical profile can be established during charge and discharge. The APTOX cell is implemented and tested both at a synchrotron facility and using an in-house diffractometer with an Ag *K*α_1_X-ray source. Furthermore, the applicability of the APTOX cell has already been demonstrated in a recent study on an all-solid-state lithium battery, Li|LiBH_4_·½CH_3_NH_2_|TiS_2_ (Grinderslev *et al.*, 2023 ▸).

## 2. The APTOX cell

The new sample environment for diffraction experiments, APTOX, has been developed to facilitate *operando* investigation of SSBs. The APTOX cell has an integrated heating element with temperature monitoring and allows for application of mechanical pressure on the battery stack. Two variants of the cell have been designed: the first variant, APTOX-Pmon, enables monitoring of the stack pressure of the cell as a function of time. Thus, pressure-induced effects during battery cycling can be monitored and compared with diffraction and electrochemical data. A simpler variant, APTOX-Spring, uses springs to sustain a constant mechanical pressure, similarly to recent constant-pressure setups described in the literature (Ham *et al.*, 2023 ▸; Lee *et al.*, 2024 ▸). The APTOX-Spring variant does not allow for monitoring of the stack pressure. The two versions of the APTOX sample environment offer versatile solutions for the *operando* investigation of mechanical, electrochemical and structural properties of SSBs. A full overview of components used for the APTOX cell can be found in the supporting information (chapter 1, Fig. S1).

### 2.1. Cell architecture and design

The body of the APTOX cell is identical for both APTOX-Pmon and APTOX-Spring. The outer diameter of the cell is 38.1 mm, which is similar to the well-known AMPIX cell (Borkiewicz *et al.*, 2012 ▸); the height of the cell (including the pressure system of choice) is 55 mm.

#### 2.1.1. Cell materials

The cell is built from two main materials, polyether­ether­ketone (PEEK 1000) and stainless steel (316-series). With a continuous service temperature up to 250 °C, PEEK is used for electrical insulation to minimize electronic noise, ensuring high-quality electrochemical data, and to avoid short-circuit of the cell. The remainder of the cell is built from 316-series stainless steel. The dimensions of the mentioned parts can be found in the step file provided as supporting information.

#### 2.1.2. Cell body

**Figure 1:**
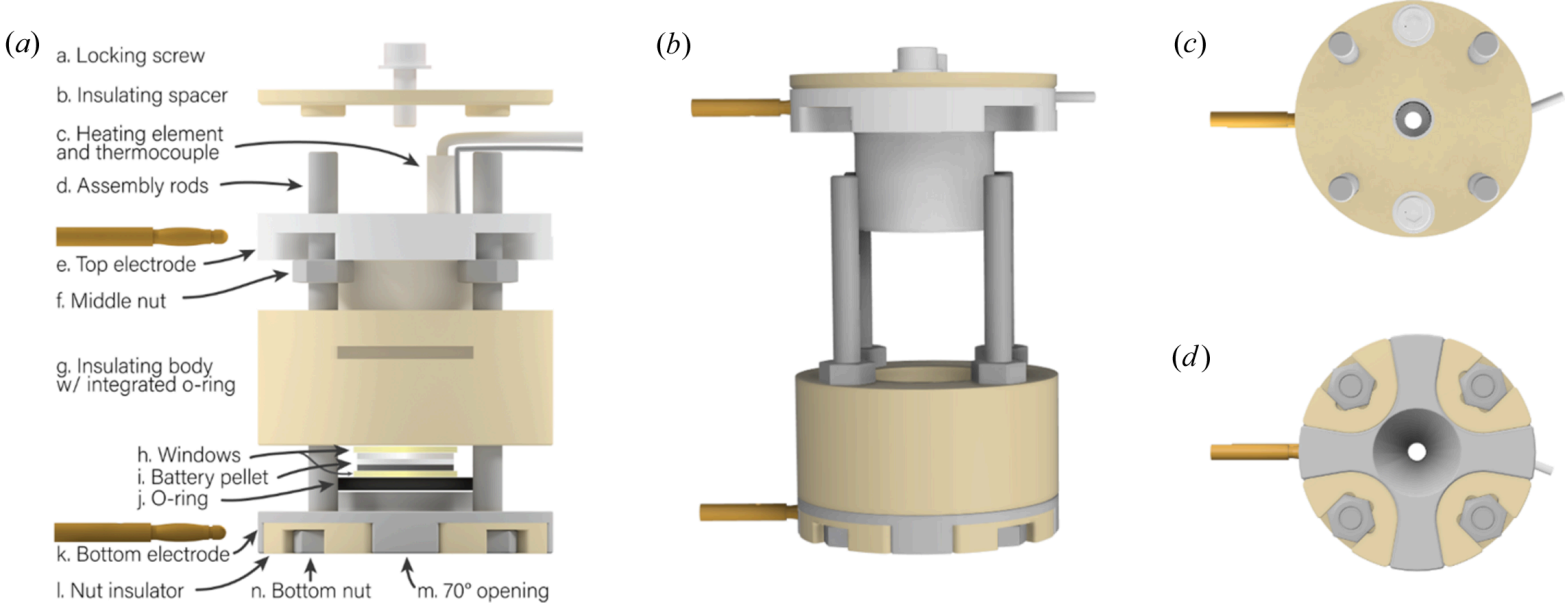
Illustration of the APTOX cell body. (*a*) Exploded view, (*b*) bottom and top part when assembled, (*c*) top view, (*d*) bottom view.

The body of the APTOX cell, Fig. 1 ▸(*a*), is held together by four long assembly rods (machine screws) (d). These are mounted to the bottom electrode (k) using bottom nuts (n). A PEEK nut insulator (l) is used to electrically insulate the assembly rods from the bottom electrode. The bottom electrode has a 70° indent for the diffracted X-rays to exit the cell (m). The insulating body (g) is placed on top of the bottom electrode, and an O-ring (j) on the bottom electrode ensures a hermetic (airtight) seal. The insulating body has a groove towards the top where another O-ring is placed to ensure a hermetic seal when the top electrode is inserted. The bottom electrode and the insulating body are held in place using the middle nuts (f), such that they constitute a single part [see Fig. 1 ▸(*b*)].

The top electrode (e) contains a groove to fit the heating element and the thermocouple (c) used to control and monitor the temperature of the cell. An insulating spacer (b) with raised edges is mounted on top to ensure electrical insulation. Two locking screws (a) are used to secure the insulating spacer. The assembled top part is shown in Fig. 1 ▸(*b*). The assembly procedure of the cell and subsequent handling of the battery pellet to assemble the electrochemical system are given in supporting information chapter 2 (Figs. S2–S9) and chapter 3. After assembling the body of the APTOX cell, either the APTOX-Pmon or the APTOX-Spring top section (described in Sections 2.1.3 and 2.1.4, respectively) can be placed on the top electrode to ensure the mechanical pressure of choice.

#### 2.1.3. APTOX-Pmon

**Figure 2:**
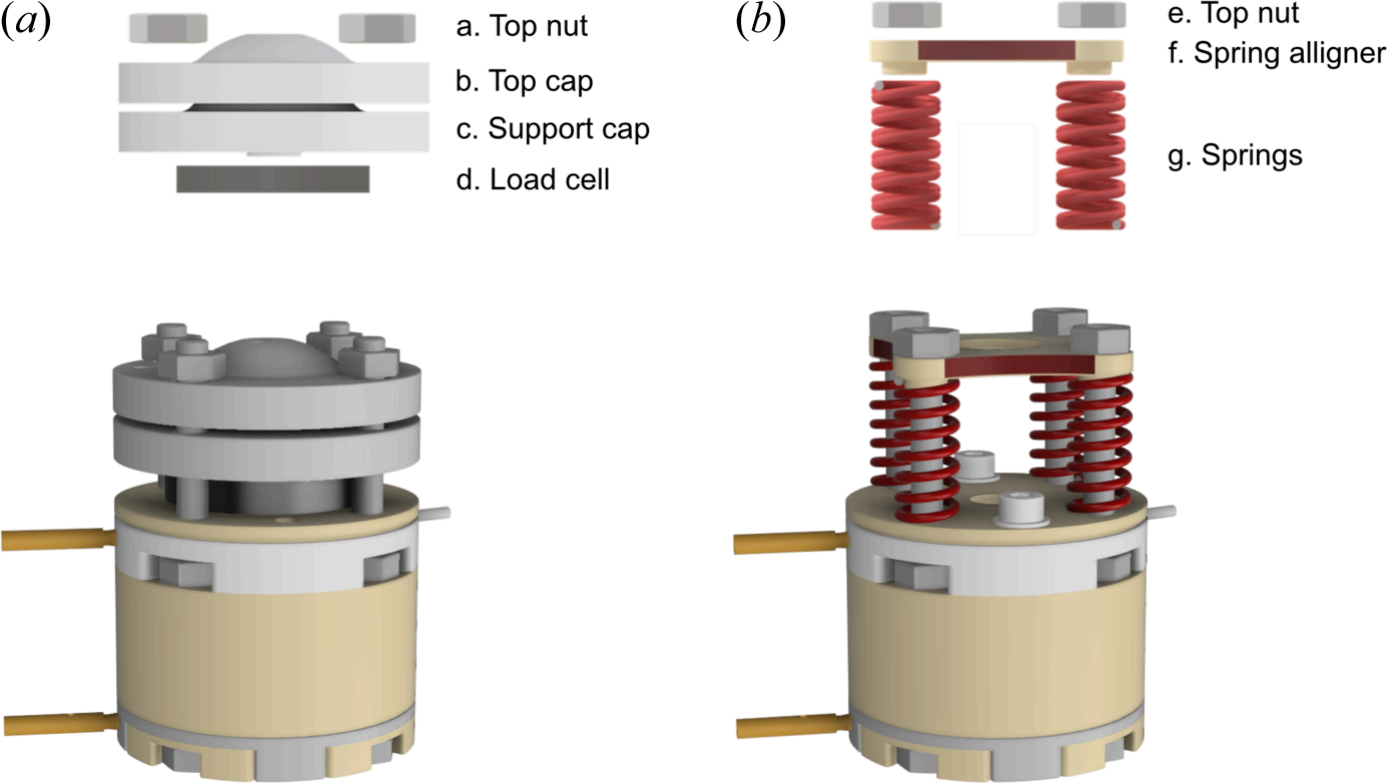
Illustration of the (*a*) APTOX-Pmon cell (top: exploded view of the pressure components; bottom: assembled APTOX-Pmon cell) and (*b*) APTOX-Spring cell (top: exploded view of the pressure components; bottom: assembled APTOX-Spring cell).

The APTOX-Pmon, illustrated in Fig. 2 ▸(*a*), enables time-resolved pressure monitoring. The two locking screws from the APTOX cell body must be removed; then a load cell can be placed on top of the insulation spacer. A top cap and a support cap (b and c) are placed on top of the load cell (d). Pressure can be applied to the top cap with four top nuts (a), which distribute the pressure uniformly on the support cap due to their hemispherical shape. Details of the electronic system monitoring the pressure are given in Section 2.3.

#### 2.1.4. APTOX-Spring

The APTOX-Spring variant, illustrated in Fig. 2 ▸(*b*), offers a simpler design using springs to ensure a stable applied mechanical pressure but does not allow for pressure monitoring. A spring (g) is placed on each assembly rod (four springs in total). A PEEK spring alignment plate (f) is placed on top of the springs and a set pressure can be applied by compressing the springs using the tightening nuts (e). A pressure frame (described in supporting information chapter 4, see Fig. S10) has been designed with the purpose of setting a specific pressure using the springs. The desired stack pressure will determine the springs needed. We have used commercially available helical compression springs with forces from 52 to 104 N per spring [*i.e.* 208–416 N (∼21–42 kgf) distributed on the four springs] (see Table S1).

### 2.2. X-ray windows

The APTOX cell has a 35° 2θ opening angle on the X-ray exiting side suitable for PXRD measurements. The opening has a diameter of 3.4 mm. The X-ray windows, stacked between the top and bottom electrode [Fig. 1 ▸(*a*)], can be glued onto the electrodes using silver ep­oxy or similar, to ensure electronic contact between the electrodes and the windows and provide an airtight seal. The windows must be rigid and strong enough to not bend or crack under the desired stack pressure. Furthermore, the windows should be airtight and keep out moisture from the sample as well as being electrically conductive. The windows can have a thickness of 0.5 to 2 mm and should be 10–18 mm in diameter. A variety of different X-ray window materials have been investigated (see Table 1).

### 2.3. Cell control and monitoring

A microcontroller monitors the stack pressure and the temperature; the controller is described in supporting information chapter 5, Fig. S11. The Teensy 3.5 microcontroller is configured as a device in a controller/device system, where a computer is the controller. On the computer, an ASCII terminal program is run which, using ASCII commands, controls the cell temperature and monitors the cell pressure. Different ASCII terminal programs can be used, *e.g.* the built-in serial monitor in the *Arduino IDE* (*Serial Monitor*) or *CoolTerm* which exists for many computer platforms. Several cells can be controlled simultaneously, by creating several parallel systems, each with its own USB connection.

#### 2.3.1. Temperature control and monitoring

A standard 4 × 15 mm cartridge heater (24 V, 35 W) is used to obtain the desired temperature. The heating cartridge of choice is typically used for UltiMaker 2 3D printers (UM2 heater cartridge upgrade 24 V 35 W 4 × 15 mm). UltiMaker 2 has a maximum operational temperature of 260 °C, but other cell components usually limit this to lower temperatures, *e.g.* PEEK 1000 (250 °C), O-rings (∼200 °C for fluorocarbon elastomer) and silver ep­oxy glue (∼150 °C).

The heater is controlled by a pulse width modulation (PWM) signal via a power transistor, in this case a MOSFET transistor. The MOSFET transistor also functions as a switch. A thermocouple (type K), placed 2 mm from the heating element, ensures the APTOX cell reaches the desired temperature. The thermocouple measures the temperature via a thermocouple amplifier (MAX31856). The thermocouple amplifier converts the analogue signal to a digital signal.

The thermocouple amplifier and the PWM signal are controlled and generated by a microcontroller using a proportional-integral-derivative (PID) regulator algorithm. In this case a Teensy 3.5 microcontroller is used. The thermocouple amplifier uses the serial peripheral interface (SPI) communication protocol.

#### 2.3.2. Pressure monitoring (APTOX-Pmon)

For the APTOX-Pmon cell, the pressure is measured by a Wheatstone bridge based load cell (LCM901-6-10KN, maximum capacity load ∼1000 kgf) via a load cell amplifier (HX711), which serves to both amplify the signal and convert the analogue signal from the load cell to a digital signal. The HX711 amplifier is a 24-bit precision analogue-to-digital converter. The digital signal is then read by the Teensy 3.5 microcontroller using the SPI communication protocol. While the load cell has a maximum capacity of ∼1000 kgf, the APTOX cell may experience issues when subjected to pressure above 981 N (∼100 kgf). The selected X-ray windows should be able to withstand the applied pressure.

## 3. Results and discussion

### 3.1. Operational metrics and cell performance

**Figure 3:**
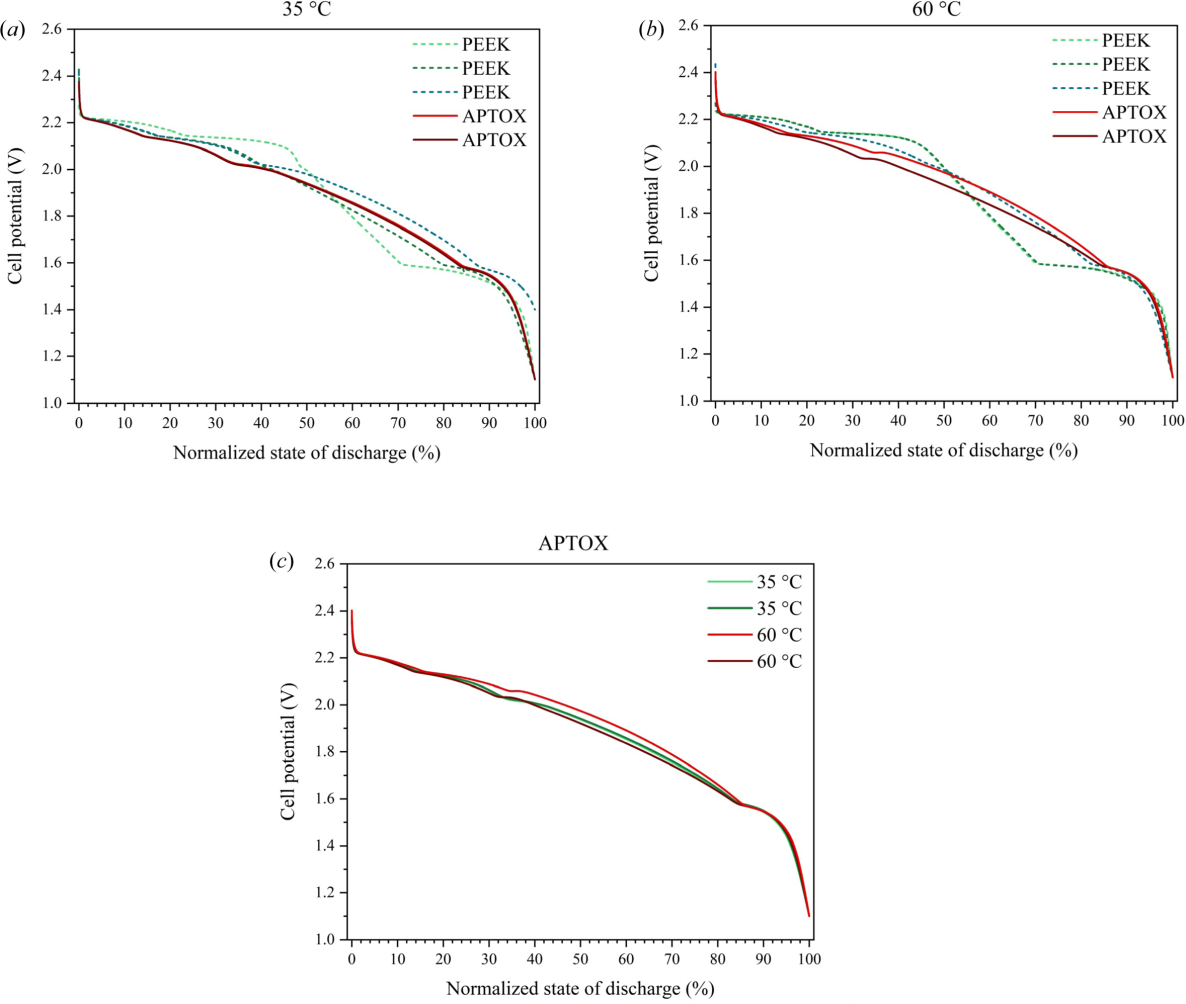
Discharge profiles of Na|Na(CB_8_H_9_)_0.04_(CB_9_H_10_)_0.96_|TiS_2_ cells in either APTOX (solid lines) or a custom-built PEEK cell (dashed lines) at (*a*) 35 °C and (*b*) 60 °C. (*c*) Comparison of discharge curves from APTOX for both temperatures. The discharge profiles are normalized. Discharge capacities can be found in Table S2 (supporting information).

To evaluate the electrochemical performance of the APTOX cell, discharges of Na|Na(CB_8_H_9_)_0.04_(CB_9_H_10_)_0.96_|TiS_2_ cells were tested in both the APTOX cell and a custom-built in-house setup made from PEEK for comparison of the electrochemical data. Details on the assembly procedures can be found in supporting information chapter 6. The tests were conducted at two different temperatures, *i.e.* 35  and 60 °C [see Figs. 3 ▸(*a*) and 3 ▸(*b*), which contain experimental data from three PEEK cells and two APTOX cells]. The cells were discharged to 1.1 V versus Na^+^/Na at a C rate of C/10, such that 10 h of discharge corresponds to the formation of NaTiS_2_. The discharge curves of all cells have been normalized such that the capacity of each individual cell is disregarded; however, the discharge capacities are provided in Table S2. For the discharge curves measured at 35 °C, the APTOX and the PEEK cells exhibit similar discharge profiles, with just one PEEK cell showing slightly different sloping behaviour (light green). The discharge curves of the APTOX cells measured at 60 °C have similar potential profiles, with two PEEK cells exhibiting different sloping behaviour (light green and dark green). All four discharge curves (*i.e.* measured at 35 and 60 °C) from the APTOX cell are shown in Fig. 3 ▸(*c*); they exhibit only minor differences. The origin of the differences observed in some of the discharge profiles of the investigated electrochemical cells needs further attention. These discrepancies may originate from minor differences in the composition of electrolytes and electrodes as well as the procedures for battery cell assembly, emphasizing the need for combined, simultaneous use of diffraction techniques, electrochemical measurements, and monitoring of stack pressure and temperature.

The frequency response of the APTOX cell has been assessed by electrochemical impedance spectroscopy (EIS) (Fig. S13). The maximum measurable impedance of the APTOX cell is ∼1 × 10^8^ Ω, while the lowest measurable impedance is ∼3 × 10^−1^ Ω at frequencies below ∼1 × 10^5^ Hz. Furthermore, the impedance of a test load of 100 Ω was reliably measured at frequencies up to ∼1.1 MHz. This allows the cell to be used for *operando* EIS investigations.

**Figure 4:**
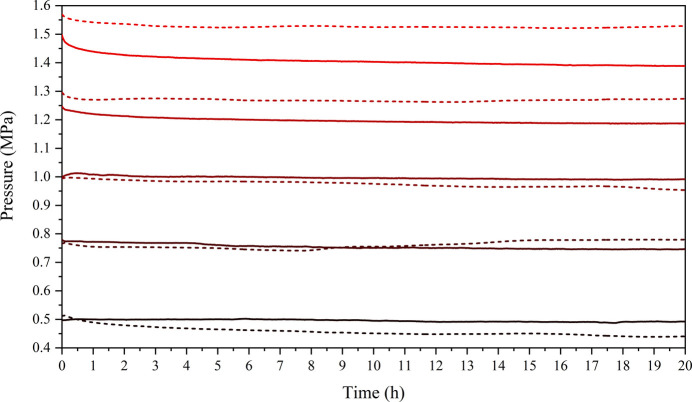
Mechanical relaxation measured using an APTOX-Pmon cell at room temperature (dashed lines) and at 60 °C (solid lines) at pressures around 0.5, 0.75, 1.0, 1.25 and 1.5 MPa. Two aluminium alloy windows were used (EN AW-5754, each 2 mm thick, Ø18 mm).

The mechanical relaxation of an empty APTOX-Pmon cell with two aluminium alloy windows (EN AW-5754) was measured for a series of applied pressures over a 20 h period at room temperature and at 60 °C (Fig. 4 ▸). Upon application of mechanical pressure, the cell quickly stabilizes, resulting in a stable mechanical pressure. To ensure the optimal pressure conditions, we recommend re-applying the pressure after 30 min of relaxation. The measurements at room temperature were conducted without temperature control, and the slight variations in pressure are related to fluctuations in temperature, as shown in supporting information chapter 6, Fig. S14.

### 3.2. X-ray windows

**Figure 5:**
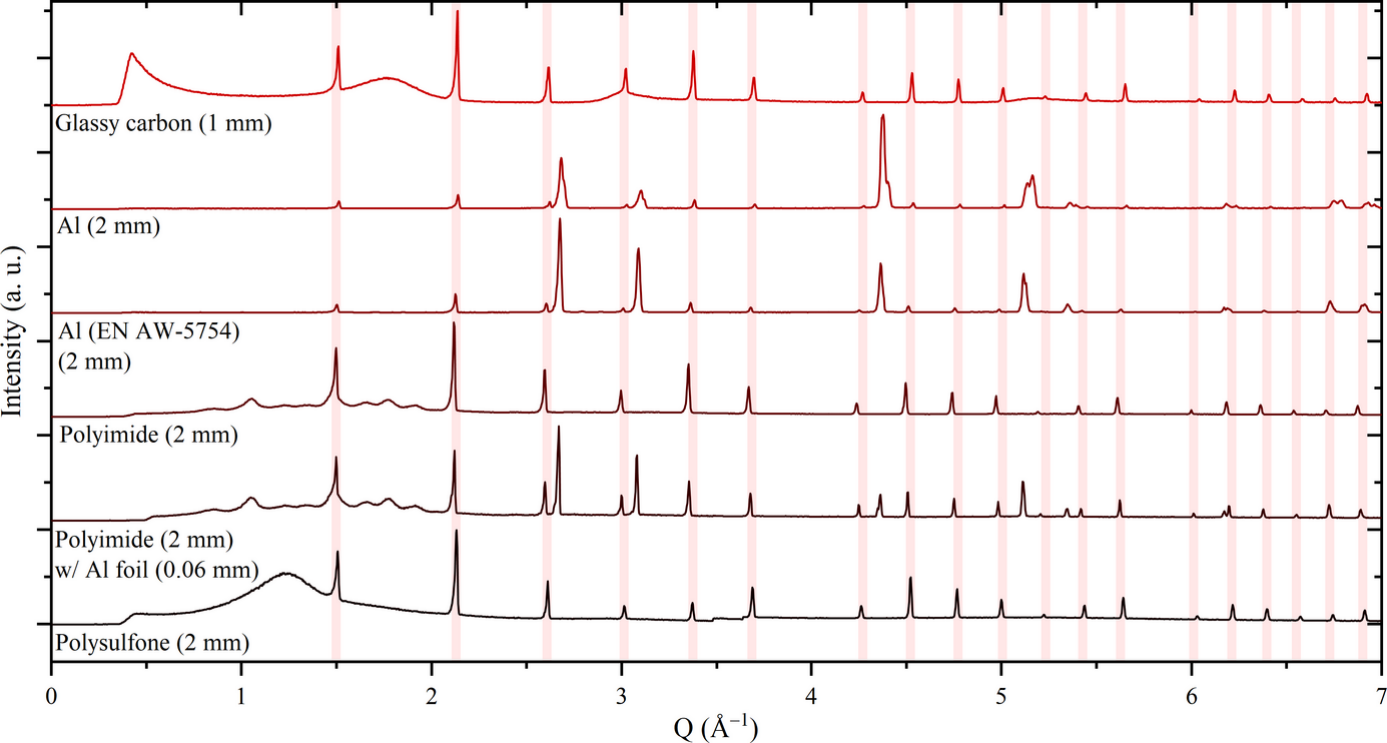
Diffractograms of LaB_6_ (marked with red lines) with different window configurations measured in-house (Ag *K*α_1_, λ = 0.5594 Å, ambient con­ditions). The intensities have been normalized.

PXRD measurements of different window configurations were conducted using an in-house Ag *K*α_1_X-ray diffractometer (Fig. 5 ▸ and Fig. S15) and at the DanMAX beamline at the MAX IV synchrotron facility (Fig. S16). Choosing a suitable window configuration is important to optimize the diffraction signal from the sample and to avoid overlapping reflections from the sample and the X-ray window, and also to minimize attenuation of the X-ray beam. To assess the viability of the windows, a thin sample of LaB_6_ mounted between two pieces of Kapton tape was measured with different window configurations.

The X-ray windows selected for this investigation are categorized into three groups: glasses, metals and polymers. Table 1 ▸ presents the physical properties and observations derived from the diffractograms obtained for the investigated X-ray windows. As the glassy materials are inherently amorphous, they cause no high-intensity Bragg reflections in the diffractograms, making them advantageous in this regard. They typically have broad reflections of low intensity. These broad reflections cause a low signal-to-background ratio (3–12%); however, high-intensity reflections of interest are easily visible in the obtained diffractograms. The main disadvantage for the glassy materials is their brittle behaviour, which may cause them to crack under pressure (Warlimont & Mar­tienssen, 2018 ▸). Additionally, borosilicate and quartz have low electronic conductivity, necessitating covering the window with an electronically conducting foil.

Metal windows, such as Al and Al alloys, are advantageous in terms of electronic and thermal conductivity as well as mechanical rigidity. These windows facilitate efficient conduction of electrons from the battery pellet to the electrodes of the APTOX cell, allowing easy implementation. A drawback of metal windows is their intense Bragg reflections in the obtained diffractograms. Significant Bragg reflections from the materials under investigation can thus be hidden in these large reflections from the metallic X-ray windows. Moreover, the high flux at synchrotron facilities can cause challenges regarding overexposure of the detector due to significant scattering from the metallic X-ray window, necessitating attenuation of the X-ray beam. Aluminium alloys display better mechanical properties than pure Al, but they also show additional weak diffraction peaks from the alloying elements.

Plastic X-ray windows, such as polyimide and polysulfone, have several broad reflections of low intensity, which causes a low signal-to-noise ratio. However, they also contain light elements, resulting in low attenuation of the X-ray beam. The plastics offer a distinct advantage over glassy materials by deforming under pressure rather than cracking. They are not electronically conducting, necessitating the use of conducting foils such as Al tape or Cu tape. This layer should be as thin as possible in order to limit scattering. Note that the chemical stability of plastics should also be considered, *e.g.* for cell cleaning, as plastics such as polysulfone are not stable in contact with acetone. Therefore, the more expensive but also more chemically resistant polyimide may be a better choice. Eventually, the ideal window configuration depends on the requirements and specifications for the system of interest.

### 3.3. *Operando* solid-state battery characterization

Two proof-of-concept battery cells were cycled (synthesis procedures and cell configurations are described in the sup­porting information, chapter 8 and Table S3). The APTOX-Spring is demonstrated at a synchrotron facility, while the APTOX-Pmon is demonstrated using an in-house diffractometer equipped with an Ag *K*α_1_X-ray source. The configurations of the battery cells, Na|Na_4_(B_12_H_12_)(B_10_H_10_)|NaCrO_2_(APTOX-Spring) and Na|Na(CB_8_H_9_)_0.04_(CB_9_H_10_)_0.96_|TiS_2_ (APTOX-Pmon), have been chosen because the cathode active materials and/or solid electrolytes are known from the literature (Jakobsen *et al.*, 2022 ▸; Meng *et al.*, 2023 ▸; Kubota *et al.*, 2015 ▸; Asakura *et al.*, 2021 ▸; Duchêne *et al.*, 2017 ▸; Grinderslev *et al.*, 2025 ▸; Chianelli *et al.*, 1975 ▸; Wiedemann *et al.*, 2019 ▸; Lin *et al.*, 2020 ▸). The investigated electrochemical cell chemistries are selected for demonstration of the new *operando* cells. The presented data reveal that further in-depth studies are needed to fully understand the complexity of the solid-state electrochemical cells.

#### 3.3.1. APTOX-Spring

**Figure 6:**
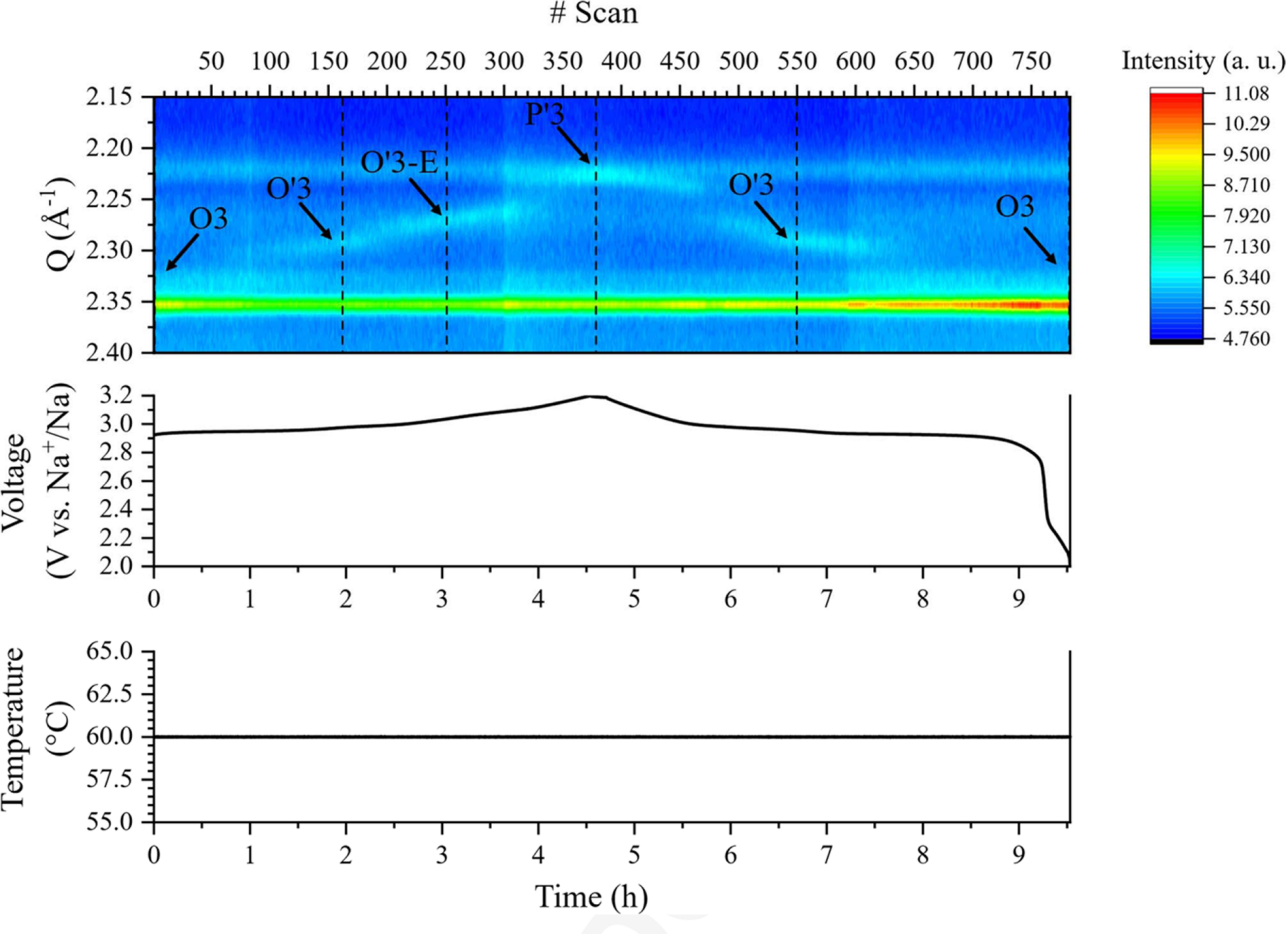
Contour plot of the collected SR-PXRD data of a precycled Na|Na_4_(B_12_H_12_)(B_10_H_10_)|NaCrO_2_ cell and the corresponding galvanostatic charge/discharge data collected between 2.0 V and 3.2 V over ∼9 h with a C rate of C/10. The dotted vertical lines show the scans used for subsequent Rietveld refinements.

The APTOX-Spring cell was used for *operando* investigation at the DanMAX beamline at the MAX IV synchrotron facility using a wavelength of λ = 0.354241 Å. The cell used four helical compression springs (STEINEL, SZ8113) applying a stack pressure of ∼1 MPa. Windows of size 2 × 2 mm Al alloy (EN AW-5754) were used, along with Cu foil as the current collector on the anode side, and the cell was cycled at 60 °C. Each diffractogram has an acquisition time of 1 s. The battery cell, Na|Na_4_(B_12_H_12_)(B_10_H_10_)|NaCrO_2_, was assembled using a Na electrode (Ø10 mm), a Na_4_(B_12_H_12_)(B_10_H_10_) electrolyte pellet (Ø10 mm, ∼60 mg), and a cathode composite electrode consisting of Na_4_(B_12_H_12_)(B_10_H_10_), NaCrO_2_ and carbon (Ø6 mm, ∼2 mg), as outlined in the supporting information, chapter 8. The smaller diameter of the cathode electrode was used to enhance the relative signal from the cathode active material. In this case, the sodium foil had the same diameter as the electrolyte pellet, but a slightly smaller diameter can be chosen to ease the cell assembly and ensure separation of the electrodes. Furthermore, the sodium metal should be rolled as thin as possible to reduce its scattering contribution. The cell was precycled for one full cycle before the *operando* measurement to ensure the battery cell could satisfactorily charge and discharge. The cell was cycled with a C/10 rate [0.5 Na^+^ in 10 h, giving a theoretical capacity of 125 mAh g^−1^ for NaCrO_2_ (Sawicki *et al.*, 2017 ▸)] with a discharge capacity of 60.4 mAh g^−1^. The C-rate did not account for an impurity of Cr_2_O_3_ (35 wt%) in the cathode active material. Adjusting for this impurity, the cell exhibited an actual discharge capacity of 92.9 mAh g^−1^. A selected *Q* range of the SR-PXRD data is shown in Fig. 6 ▸, highlighting the peaks from the O3, O′3, O′3-E and P′3 phases of Na*_x_*CrO_2_. The evolution of the phases during charge and discharge is in agreement with previous studies and the lattice parameters of the individual phases were successfully determined using Rietveld refinement (Table 2 ▸) (Jakobsen *et al.*, 2022 ▸). The signal-to-background ratio is low for the relevant diffraction peaks from Na*_x_*CrO_2_ in the *Q* range 2.20–2.35 Å^−1^. This is due to both the small amount of the cathode active material and significant scattering from several components positioned in the X-ray beam, *i.e.* the X-ray windows, Cu foil, electrolyte and sodium metal. Ensuring a minimum overlap between the cathode active materials and the remaining components is essential for obtaining good results. The temperature of the cell was stable at 60 °C throughout the cycle. The full *Q* range of diffraction data is shown in Fig. S17. The apparent peak splitting of the Bragg peaks corresponding to the Al windows is due to their different zero offset.

An APTOX-Spring cell with a similar window configuration has previously been used for a Li|LiBH_4_·½CH_3_NH_2_|TiS_2_SSB cell, also measured at the DanMAX beamline (λ = 0.354241 Å) (Grinderslev *et al.*, 2023 ▸). The cell used four helical compression springs (STEINEL, SZ8113) with a total spring load of 1.25 MPa, and the measurement was conducted at room temperature, without the use of temperature control. Sequential Rietveld refinement was successfully performed, showing the evolution of the unit-cell parameters of the cathode active material, TiS_2_, during battery cycling (Grinderslev *et al.*, 2023 ▸).

#### 3.3.2. APTOX-Pmon

**Figure 7:**
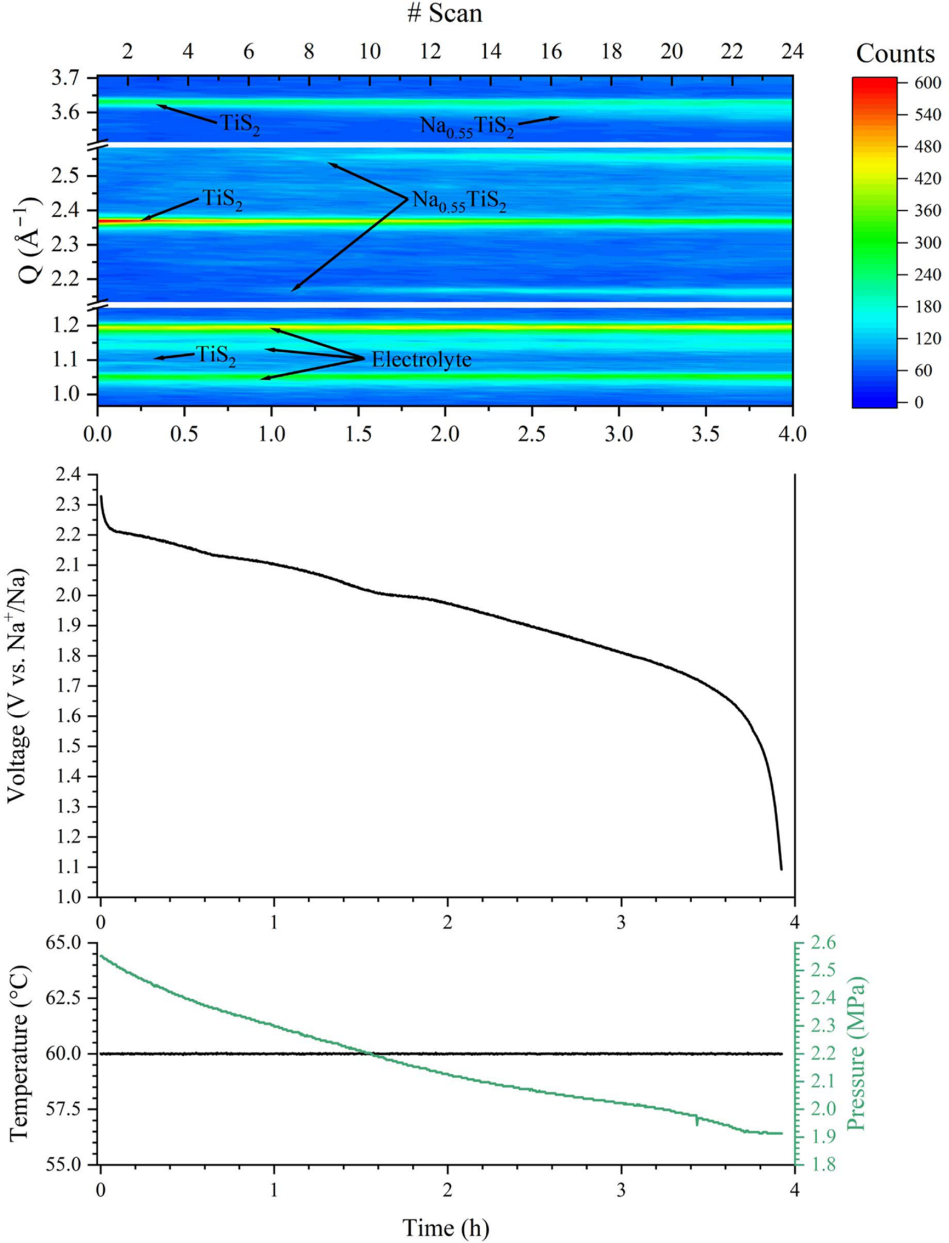
Contour plot of the collected in-house PXRD data of a Na|Na(CB_8_H_9_)_0.04_(CB_9_H_10_)_0.96_|TiS_2_ cell and the corresponding galvanostatic discharge data collected between 2.3 and 1.1 V. The cell was run with a C-rate of C/15. The bottom plot shows the temperature of the cell along with the measured pressure. An APTOX-Pmon cell was used.

The APTOX-Pmon cell was demonstrated by *operando* investigation using in-house facilities (Ag *K*α_1_, λ = 0.5594 Å) with a stack pressure of ∼2.5 MPa at 60 °C and using 1 × 0.5 mm and 1 × 2 mm Al-alloy (EN AW-5754) windows. Each diffractogram has an acquisition time of 10 min. A Na|Na(CB_8_H_9_)_0.04_(CB_9_H_10_)_0.96_|TiS_2_ cell was discharged at a C/15 rate, such that 15 h of discharge forms NaTiS_2_. Three different segments in *Q* space of the PXRD data are presented in Fig. 7 ▸, showing the evolution of the Bragg reflections corre­sponding to the transition from TiS_2_ (*P*3*m*1) to Na_0.55_TiS_2_ (*R*3*m*) during discharge (Table 3 ▸). Bragg diffraction peaks are observed from all the cell components, *i.e.* the Na electrode, the Cu-current collector and the Na(CB_8_H_9_)_0.04_­(CB_9_H_10_)_0.96_ solid electrolyte as well as the Al-alloy windows (see the full *Q* range of diffraction data in Fig. S18). The in-house setup is limited by the reduced time resolution, caused by the lower intensity as compared with synchrotron sources. This also limits the ability to average several measurements across the cell to decrease the effects of preferred orientation and improve statistics. During discharge, the stack pressure decreases from 2.55 to 1.91 MPa, plateauing towards the end of discharge. The temperature remained stable at 60 °C throughout the experiment. The discharge capacity of the cell was 62.45 mAh g^−1^. A conversion of 53.6% to Na_0.55_TiS_2_ was observed by X-ray diffraction (see Table 3 ▸ and Fig. S19). The low degree of conversion may explain the low discharge capacity. Reducing TiS_2_ particle size may resolve some of these issues as the diffraction data suggest a sluggish and incomplete reaction with some of the cathode material.

#### 3.3.3. Refinement considerations

While possible, Rietveld refinements or Le Bail fitting are mostly limited to one component per refinement, as the spatial separation between individual cell components causes a unique shift in the diffraction pattern of each component. These issues can be mitigated by selecting an appropriate window and electrolyte combination without overlapping with the cathode material. This allows for the exclusion of diffraction peaks belonging to everything other than the cathode. Alternatively, reducing the thickness of each layer will reduce these effects.

## 4. Related literature

The following references are cited only in the supporting information: Barrett (1967 ▸), Bo *et al.* (2016 ▸), Huang *et al.* (2023 ▸), Zheng *et al.* (2019 ▸).

## 5. Conclusion

The new APTOX cell allows for *operando* PXRD investigation of all-solid-state batteries and may facilitate major progress in the development of novel electrochemical systems. The APTOX cell has a built-in heating element and enables stack pressure control, allowing for optimal conditions for operation of an all-solid-state battery. The versatile APTOX cell enables a large variety of cycling conditions in a temperature range from room temperature to at least 100 °C with a stack pressure from 0 to 981 N (0–100 kgf stack pressure), making tailored *operando* experiments easy to conduct. The APTOX cell exists in two variants: APTOX-Pmon and APTOX-Spring. APTOX-Pmon facilitates monitoring of the applied stack pressure, while the APTOX-Spring uses helical compression springs to ensure a stable applied stack pressure. The applicability of the cells has been demonstrated by *operando* PXRD measurements, *i.e.* the simultaneous collection of electrochemical, temperature and PXRD data of a Na|Na_4_(B_12_H_12_)(B_10_H_10_)|NaCrO_2_ cell and a Na|Na(CB_8_H_9_)_0.04_(CB_9_H_10_)_0.96_|TiS_2_ cell, for which the stack pressure was monitored, followed by subsequent analysis of the lattice parameters of the active cathode material at different states of charge. Data have been collected both using an in-house laboratory X-ray source and at the synchrotron facility MAX IV at the DanMAX beamline.

## Supplementary Material

3D model of cell. DOI: 10.1107/S1600576726000853/iu5072sup1.txt

3D model of pressure frame. DOI: 10.1107/S1600576726000853/iu5072sup2.txt

Supporting information. DOI: 10.1107/S1600576726000853/iu5072sup3.pdf

## Figures and Tables

**Table 1:** Selected physical properties of different X-ray windows The amorphous windows display broad diffraction signals at certain *Q*-values, while the crystalline windows have sharp Bragg peaks. The X-ray attenuation was calculated using the tool at https://11bm.xray.aps.anl.gov/absorb/absorb.php (Argonne National Laboratory). The signal-to-background value is calculated as the ratio of the LaB_6_ peak area to the total area under the diffractogram for the data measured on a STOE STADI P diffractometer (λ = 0.5594 Å). All measurements were conducted using two identical windows, such that a total window thickness of 2 mm stems from usage of two 1 mm-thick windows.

Type	Material	Total window thickness (mm)	Diffraction peaks at *Q*-values (Å^−1^)	Attenuation (for Ag *K*α_1_) (%)	Signal-to-background	Young’s modulus (GPa)	Yield strength (GPa)	Tensile strength (GPa)	Electronic conductivity (S cm^−1^)	Thermal conductivity (W m^−1^ K^−1^)
Glasses	Glassy carbon (Sigradur Grade G)	1	Amorphous (1.75, 3.00, 5.17)	4	12	35*^a^*	0.48†*^a^*		222.2*^a^*	6.3*^a^*
	Borosilicate	1	Amorphous (1.53)	33	6	∼65*^b^*			1×10^−15^–1×10^−13^*^c^*	2*^c^*
	Quartz	2	Amorphous (1.50)	75	3	∼73*^d^*			1×10^−14*d*^	1.38*^d^*

Metals	Aluminium	1	Crystalline (2.68, 3.10, 4.38, 5.14, 5.37)	70	72	70.2*^d^*		0.09–0.1*^d^*	50×10^3*d*^	237*^d^*
	Aluminium	2	Crystalline (2.68, 3.10, 4.38, 5.14, 5.37)	91	58	70.2*^d^*		0.09–0.1*^d^*	50×10^3^*^d^*	237*^d^*
	Al (EN AW-5754)	2	Crystalline (2.67, 3.09, 4.36, 5.12, 5.35)	∼91	63	∼73*^d^*		∼0.2–0.3*^d^*	2.0–2.3*^d^*	140–160*^d^*

Polymers	Polyimide	2	Amorphous (0.85, 1.05, 1.23, 1.35, 1.50, 1.65, 1.77, 1.91)	11	18	3.0–3.2*^d^*			1×10^−16*d*^	0.29–0.35*^d^*
	Polysulfone	2	Amorphous (1.23)	22	9	2.5–2.7*^d^*	0.070–0.080*^d^*		1×10^−15*d*^	0.28*^d^*

(*a*) HTW Germany, https://htw-germany.com/en, (*b*) Martienssen & Warlimont (2006 ▸), (*c*) Lima *et al.* (2012 ▸), (*d*) Warlimont & Martienssen (2018 ▸).

† Yield strength is replaced by compressive strength.

**Table 2:** Lattice parameters extracted using Rietveld refinements from selected scans of the *operando* data presented in Fig. 6 ▸

#Scan	Cathode phase	Space group	*a* (Å)	*b* (Å)	*c* (Å)	β (°)	*V*/*Z* (Å^3^)
1	O3	*R* 3 *m*	2.978 (12)	–	16.04 (6)	–	41.07 (3)
162	O′3	*C*2/*m*	5.06 (13)	2.95 (4)	5.80 (7)	109 (2)	41 (1)
251	O′3-E	*C*2/*m*	5.08 (9)	2.92 (4)	5.75 (7)	106 (2)	41 (1)
378	P′3	*C*2/*m*	5.05 (6)	2.90 (3)	5.87 (4)	106 (1)	41 (1)
549	O′3	*C*2/*m*	5.06 (4)	2.94 (2)	5.81 (4)	109 (1)	41 (1)
784	O3	*R* 3 *m*	2.980 (8)	–	16.02 (4)	–	41.07 (2)

**Table 3:** Lattice parameters extracted using Rietveld refinement of selected *operando* data presented in Fig. 7 ▸ (see Fig. S19) The data in column 2, ‘State of discharge’, are estimated state of discharge at the end of the scan.

#Scan	State of discharge (%)	Cathode phase	Space group	*a* (Å)	*c* (Å)	*V*/*Z* (Å^3^)	Composition
1	1	TiS_2_	*P*3*m*1	3.443 (2)	5.619 (1)	57.69 (2)	100%
13	14	TiS_2_	*P*3*m*1	3.448 (1)	5.597 (3)	57.62 (3)	72.77%
		Na_0.55_TiS_2_	*R*3*m*	3.443 (1)	21.23 (1)	72.67 (7)	27.23%
24	26	TiS_2_	*P*3*m*1	3.446 (1)	5.605 (3)	57.67 (3)	46.40%
		Na_0.55_TiS_2_	*R*3*m*	3.468 (1)	21.02 (1)	73.00 (5)	53.60%
