# Supplementary material for: Sample environment for operando solid-state battery characterization
Source: J Appl Crystallogr. 2026 Mar 20;59(Pt 2):513–23. doi: 10.1107/S1600576726000853 (PMC13060469; doi:10.1107/S1600576726000853)
Supplement: Supplementary file 3 [file j-59-00513-sup3.pdf]

JOURNAL OF APPLIED CRYSTALLOGRAPHY

Volume 59 (2026)

Supporting information for article:

Sample environment for *operando* solid-state battery characterization

Therese S. S. Faurskov, Lasse N. Skov, Jakob B. Grinderslev, Lasse G. Kristensen, Jens Magnus Horsted Bendtsen, Mads Kofod Dahl, Tommy Kessler, Bettina Pilgaard Andersen, Innokenty Kantor, Mads R. V. Jørgensen, Dorthe B. Ravnsbaek and Torben R. Jensen

Supporting information for article:

Sample environment for *operando* solid-state battery characterization

Table of Contents

Chapter 1 Illustration of the APTOX Cell

Chapter 2 Assembly Procedure of an APTOX Cell

Chapter 3 Sample Handling and Assembly of Electrochemical Cell

Chapter 4 Pressure Frame (APTOX-Spring)

Chapter 5 Cell Control and Monitoring

Chapter 6 Operational Metrics

Chapter 7 X-Ray Windows

Chapter 8 Experimental

Chapter 9 Results

Chapter 1 Illustration of the APTOX Cell

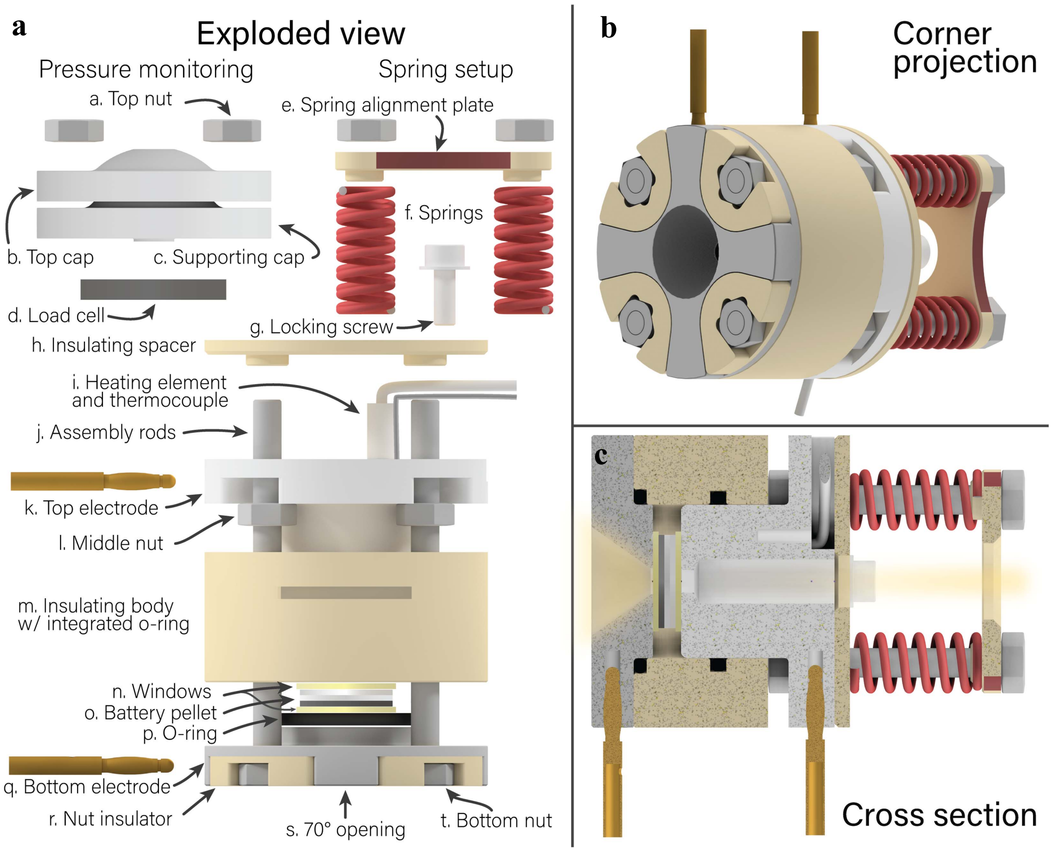

Figure S1 Illustration of the APTOX cell a) exploded view b) corner projection c) cross section.

Chapter 2 Assembly Procedure of an APTOX Cell

Step 1. Windows

Step 1 is to mount the X-ray windows. These should be 10-18 mm in diameter. In our setup we have used Ø18 mm. The height of the windows can vary as long as the top electrode is below the upper O-ring upon assembly, however, a height of 0.5-2 mm is recommended. PEEK-alignment rings can be used to center the windows as seen in Figure S2. Silver-epoxy glue, or similar, should be used to ensure electronic contact between the electrodes and the windows. Only a thin layer of glue is needed and should be well distributed. A groove has been made on each electrode to collect excess glue. A slight mechanical pressure should be applied to the window while the glue is curing to create an airtight seal.

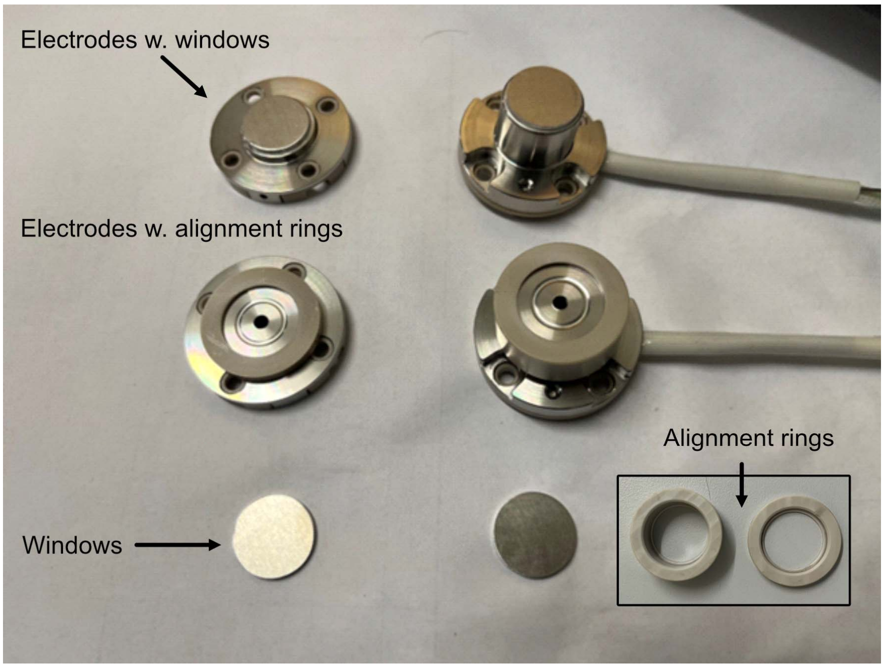

Figure S2 Top and Bottom electrodes with and without alignment rings and Al-alloy (EN AW-5754) windows.

Step 2. O-rings

A greased O-ring is placed on the bottom electrode and inside the insulating PEEK body, see Figure S3.

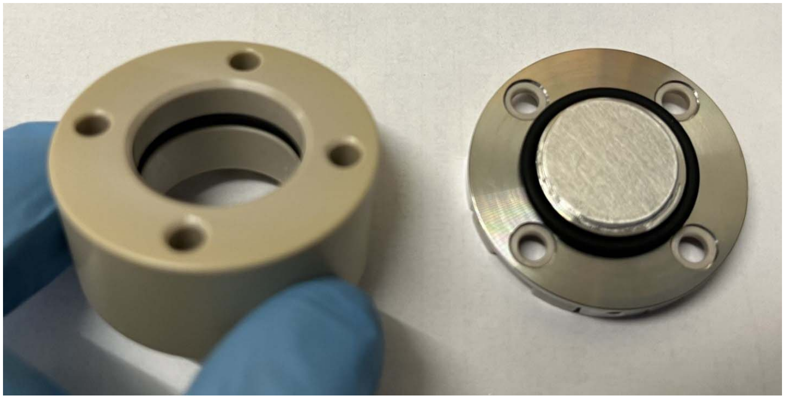

Figure S3 Placement of O-rings.

Step 3 Frame Insulation

The nut insulators must be added to the bottom electrode, as seen in Figure S4, to electrically insulate the nuts from the electrochemical cell.

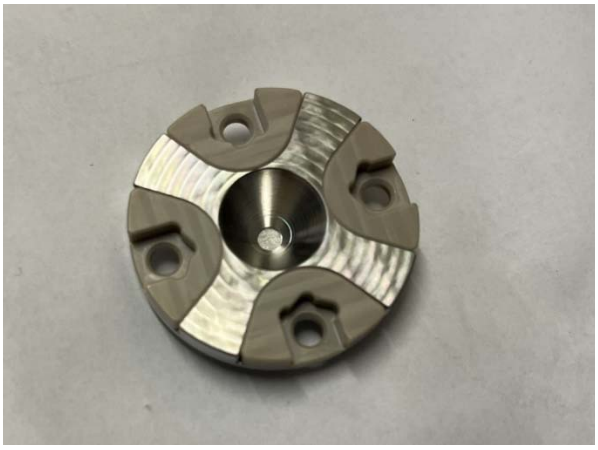

Figure S4 Nut insulators on the bottom electrode.

Step 4. Alignment of Assembly Rods

The assembly rods and the insulating PEEK body are placed on the bottom electrode (Figure S5a). The middle nuts are placed on top of the assembly rods. In order for the assembly rods to be aligned, a PEEK alignment rod is placed on the four rods after the nuts have been hand-tightened towards the O-ring, see Figure S5b. The nuts can then be gently tightened diagonally to ensure an airtight seal. The PEEK alignment rod has aligned holes to make sure the assembly rods are in the correct position, see Figure S5c.

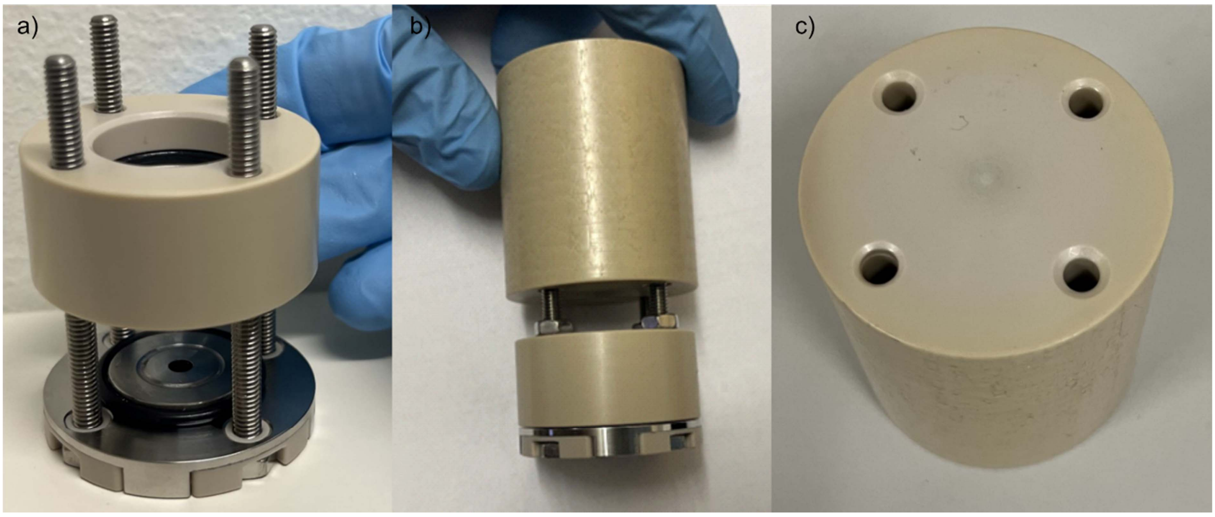

Figure S5 Assembly of cell a) bottom electrode with assembly rods end insulating body b) rod alignment c) PEEK alignment rod.

Step 5. Assembly of the Top Electrode

Place the heating element and the thermocouple in their respective grooves, see Figure S6a. Push down the insulating spacer until it clicks, see Figure S6b. If the spring system is used, use the locking screws.

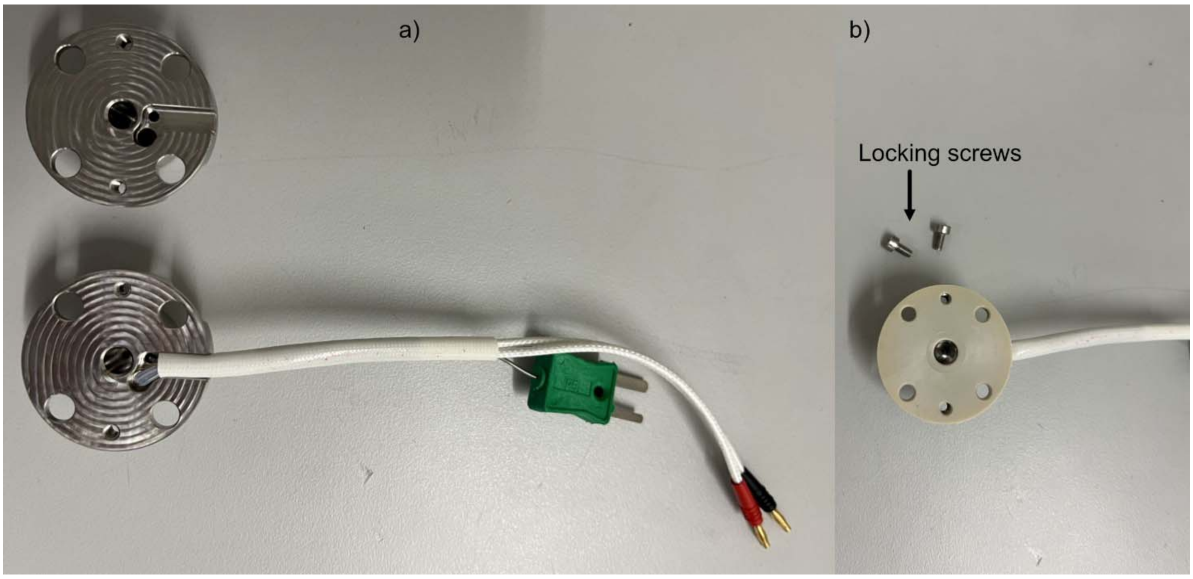

Figure S6 Top electrode a) without and with the thermocouple and heating element b) with insulating spacer.

Step 6. Sample Assembly

PEEK alignment rings or rubber rings can be used to center the battery pellet or sample of interest, see Figure S7a. This ensures the sample is centered in the X-ray beam and prevents short circuit of the battery at the pellet edge in addition to providing structural integrity to the pellet. PEEK alignment rings can be made in different heights such as 0.3 mm, 0.5 mm and 0.8 mm to match the height of the battery cell, see Figure S7b. The alignment ring should ideally be a bit lower than the battery pellet to provide structural integrity while also ensuring proper contact between the battery components and the electrodes of the *operando* cell, as well as not affecting the applied stack pressure.

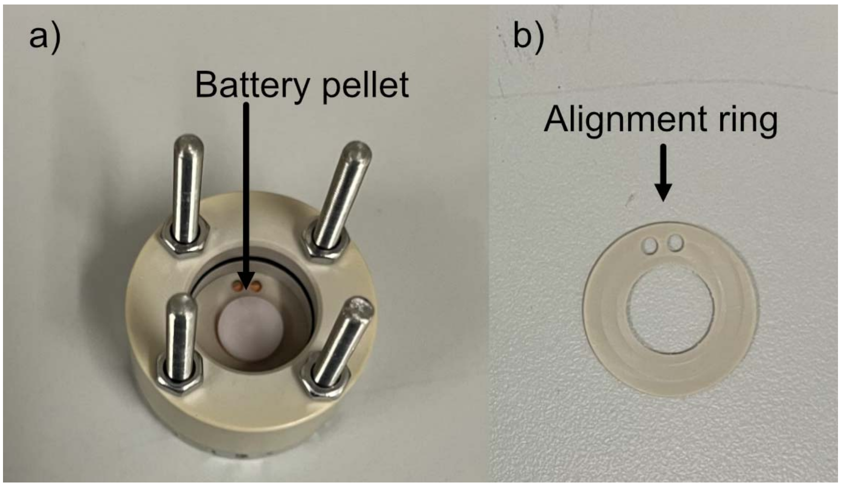

Figure S7 The lower part of the APTOX cell with battery pellet a) lower part of the APTOIX cell with Cu current collector, a PEEK alignment ring and a NaCl pellet (diameter: 10 mm) representing a battery pellet b) PEEK alignment ring.

Step 7. Place Top Electrode

Once the sample has been mounted and centered in the *operando* cell, the top electrode is mounted using the alignment rods and pushed down until you feel the tension of the O-ring, see Figure S8. Subsequently, push gently to avoid cracking your pellet until the top electrode reaches the battery pellet. The cell is now hermetically sealed and can be exported from the glovebox, and a stack pressure can now be set either using the spring system (APTOX-Spring) or the pressure distribution hemispheres (APTOX-Pmon) as seen in Figure S9.

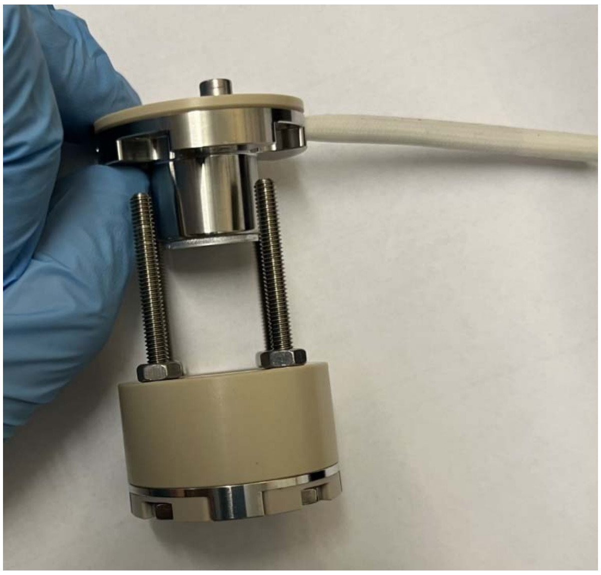

Figure S8 Sealing the APTOX cell.

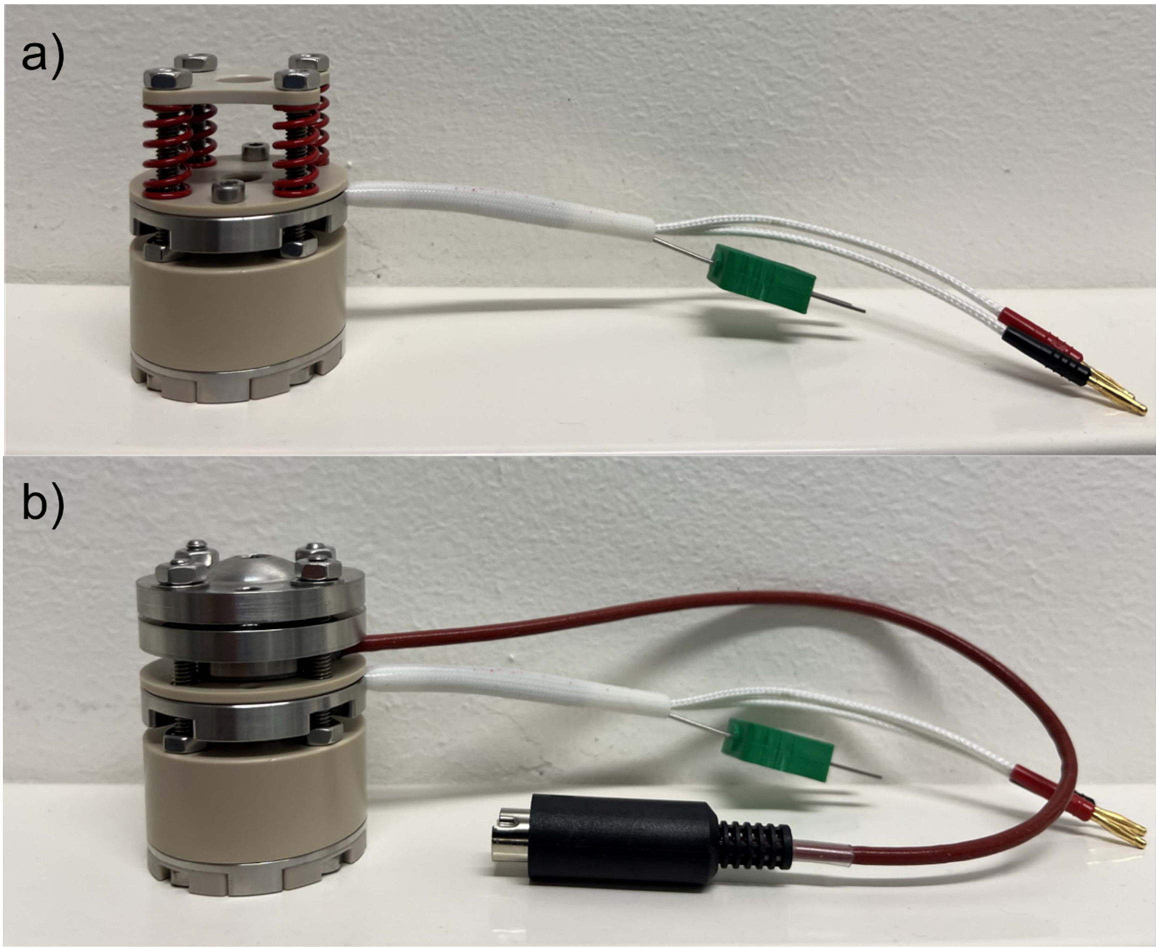

Figure S9 Assembled APTOX cells using a) the spring system (APTOX-Spring) and b) the load cell (APTOX-Pmon).

Chapter 3 Sample Handling and Assembly of Electrochemical Cell

Once the APTOX cell is built in accordance with supporting Chapter 2, an electrochemical cell can be assembled and investigated. The assembly of the APTOX cell is done from the bottom and up. As previously mentioned, the insulating body should be attached to the bottom electrode constituting a single part. The battery pellet, current collector, and any alignment rings to keep the battery stack centered can be placed on the window on the bottom electrode. The top electrode can then be pushed into the insulating body, becoming hermetically sealed using the O-ring. The cell can now be exported from a glovebox and the stack pressure can be set either using the pressure frame, Figure S10, for the spring system or using the pressure distribution hemispheres by placing the load cell, support cap and top cap and tighten the nuts to achieve the desired pressure. Lastly, the APTOX cell can be connected to a potentiostat of choice using banana-plug cables.

Chapter 4 Pressure Frame (APTOX-Spring)

A pressure frame, Figure S10, has been designed to ensure the desired stack pressure on the battery pellet when using the APTOX-spring cell. The frame consists of an aluminum frame with a load cell at the bottom. The assembled APTOX cell is then placed onto the brass alignment tool and a thrust ball bearing adapter is placed onto the spring aligner. The adapter consists of a PEEK-bottom and a metal top with a thrust ball bearing on top. The PEEK part of the adapter is placed onto the PEEK spring alignment plate to minimize the wear on the cell. The head of the screw is then turned to apply the desired stack pressure onto the cell. When the stack pressure of choice has been reached, the springs are tightened using the tightening nuts, such that the spring system maintains the applied pressure from the pressure frame.

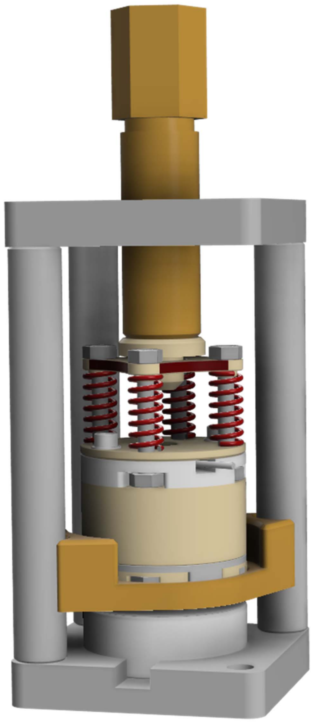

Figure S10 Pressure frame used to ensure correct stack pressure on the APTOX cell with an assembled APTOX cell placed onto the brass alignment tool.

Table S1 Technical data of springs used for APTOX-Spring.

APTOX-Spring	Type	Manufacturer	Force (N)	Length (mm)	Outer Diameter (mm)	Inner Diameter (mm)	Name
Springs for stack pressure	Helical compression spring	STEINEL	52.39	16	8	5.5	SZ8113
		STEINEL	103.93	16	8	4.8	SZ8114

Chapter 5 Cell Control and Monitoring

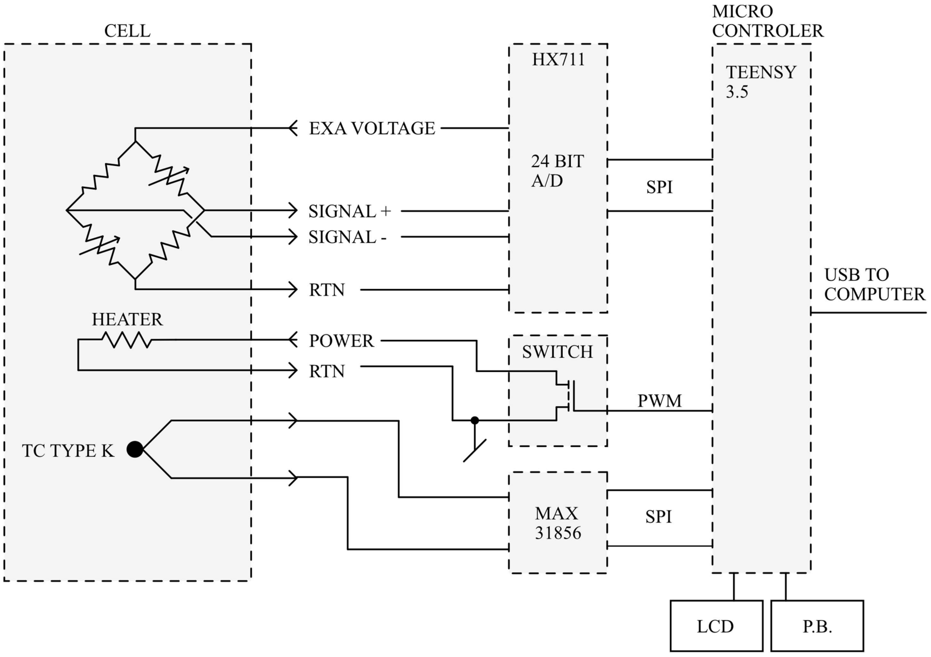

Figure S11 Diagram of proposed structure of temperature and pressure control/monitoring module. TC: thermocouple, RTN: return, EXA voltage: excitation voltage, A/D: analogue-to-digital converter, Switch: power transistor which also functions as a switch, PWM: pulse width modulation, SPI: serial peripheral interface, LCD: liquid crystal display, P.B.: push button.

Chapter 6 Operational Metrics

Table S1 Specifications for tests comparing the APTOX cell to a custom-build PEEK cell.

	APTOX	PEEK
Ø CAM-mix (mm)	5	6
Ø SE (mm)	5	10.3
Ø Na (mm)	6	10
Pelletizing pressure (MPa)	500	350
Pelletizing time (min)	3	3
Stack pressure (MPa)	1-2	1-2
CAM/area (mg/cm^2)	15	15

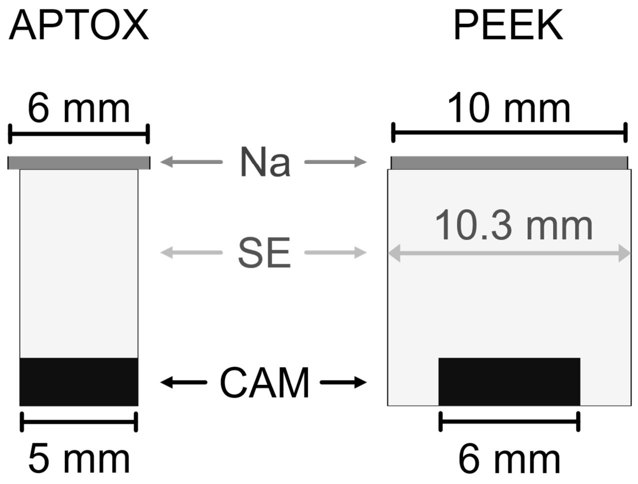

Figure S12 Illustration of the dimensions of the APTOX and PEEK cells used for the testing in Figure 3. Note the height of the pellets are not to scale. As the Na-disk (Ø 6mm) is wider than the solid electrolyte (SE) pellet (Ø 5 mm) for the APTOX cell, the Na-disk was placed at the bottom and cathode active material (CAM) mix at the top (close to the heating element) to mitigate sodium creep.

Table S2 Discharge capacities of cells used for comparison between APTOX and custom-built PEEK cells. The theoretical capacity is 240 mAh/g.

	APTOX	PEEK
	Discharge capacity (mAh/g)	Discharge capacity (mAh/g)
35 °C	191.3	226.8
	200.2	165.7
	-	239.5
60 °C	197.9	223.9
	210.4	215.8
	-	222.9

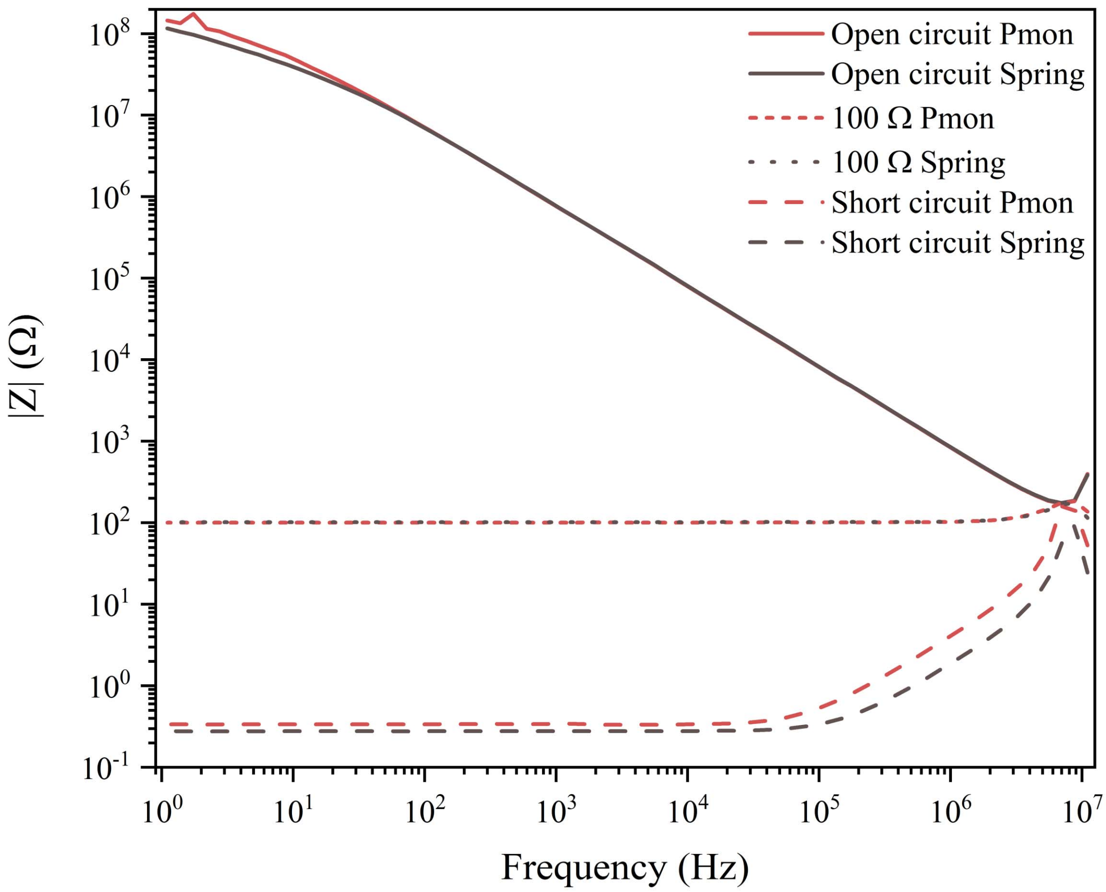

Figure S13 Open circuit, short circuit and 100 Ω load impedances of the APTOX cell using the Pmon and the Spring versions.

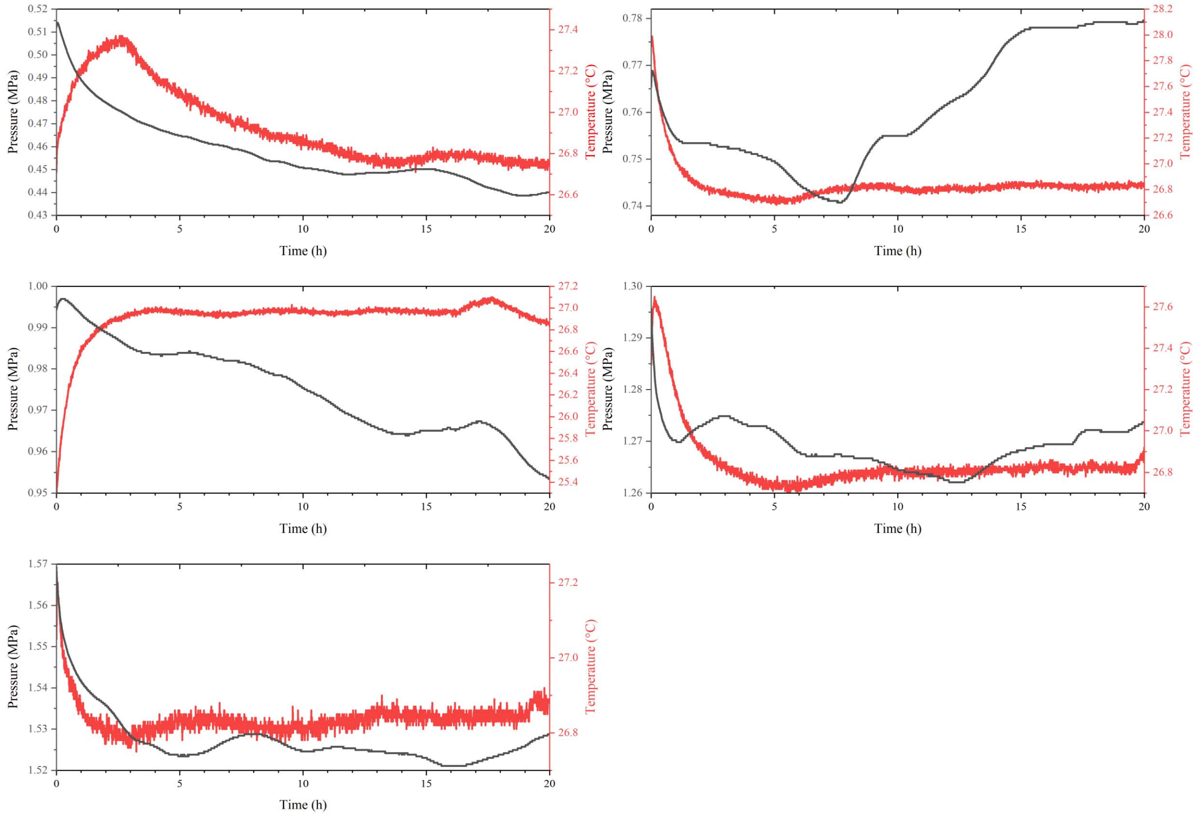

Figure S14 Mechanical relaxation of a cell with 2 x 2 mm Al-alloy (EN AW-5754) windows at different pressures with temperature monitoring. This shows that the cell initially undergoes mechanical relaxation. Subsequently, the small variations in the pressure can likely be related to changes in temperature, demonstrating the high sensitivity of the pressure monitoring. The measurements are performed at room temperature.

Chapter 7 X-Ray Windows

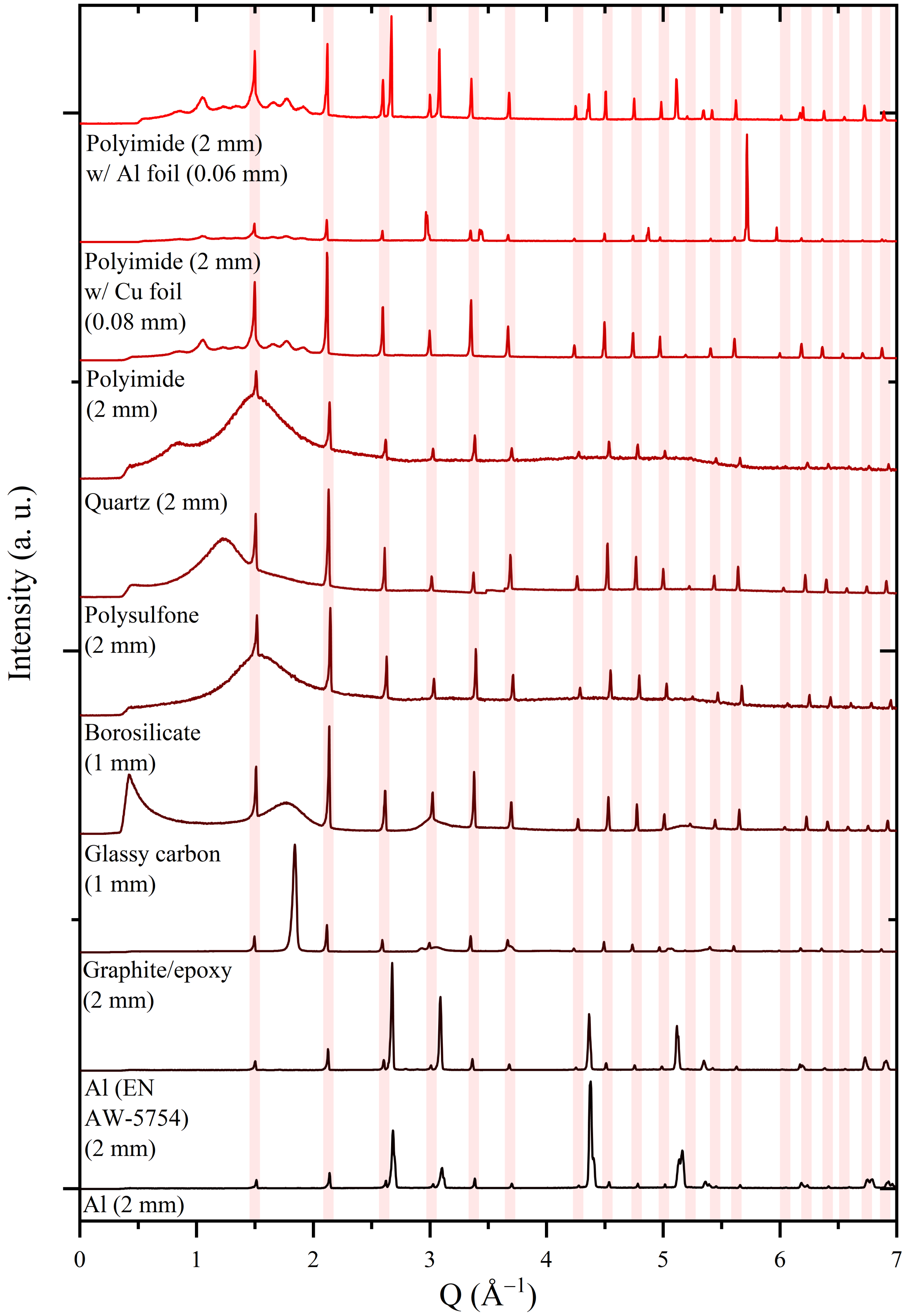

Figure S15 Diffractograms of LaB_6 (marked with red) with different window configurations measured in-house (λ = 0.5594 Å, ambient temperature). The total window thickness is given.

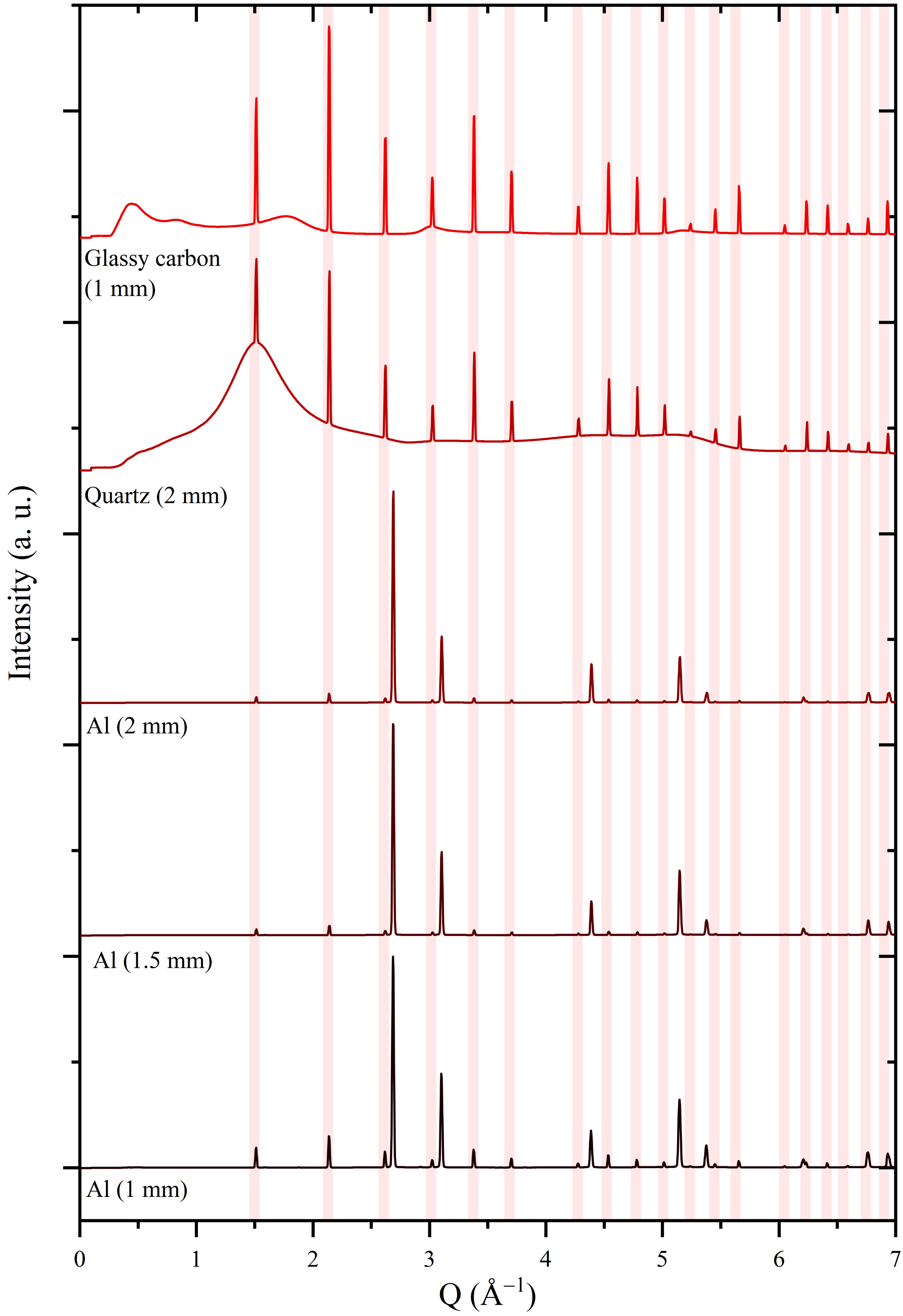

Figure S16 Diffractograms of LaB_6 (marked with red) with different window configurations at measured at the synchrotron X-ray facility DanMAX (λ = 0.354241 Å). The total window thickness is given.

Chapter 8 Experimental

Synthesis of $NaCrO_2$

$NaCrO_2$ was prepared as described by Jakobsen *et al.* (Jakobsen *et al.*, 2022). Stoichiometric amounts of Na_2CO_3 (Merck, anhydrous, 99.8%) and Cr_2O_3 (Merck, 99%) were mixed using high energy ball milling with 300 rpm spinning speed for 2 hours active ball milling. The mixed precursor was pressed into a pellet at 30 kN for 2 min. The pellet was sintered in a tube furnace with argon flow at 900 °C for 5 h and cooled to room temperature at a rate of 300 °C/h. Due to the sensitivity of the material towards air and water (Zheng *et al.*, 2019), the material was immediately transferred to an argon-filled glovebox.

Synthesis of $Na_4(B_{12}H_{12})(B_{10}H_{10})$

$Na_4(B_{12}H_{12})(B_{10}H_{10})$ was synthesized by dissolving molar equivalents of $Na_2B_{12}H_{12}$ (Katchem) and $Na_2B_{10}H_{10}$ (Katchem) in isopropylalcohol and drying the solution on a rotary evaporator at 60 °C. The electrolyte was subsequently dried at 180 °C for 16 hours under vacuum. When dry, the powder was ground by hand in a mortar for 15 minutes (Huang *et al.*, 2023).

Synthesis of $Na(CB_8H_9)_{0.04}(CB_9H_{10})_{0.96}$

$Na(CB_8H_9)_{0.04}(CB_9H_{10})_{0.96}$ was synthesized in accordance with previous published work (B. Grinderslev *et al.*, 2025).

Battery Assembly

For electrochemical measurements a cathode composite of $NaCrO_2$, $Na_4(B_{12}H_{12})(B_{10}H_{10})$ and Carbon Super C65 with a 70:20:10 weight ratio was prepared by solution impregnation described previously by (Asakura *et al.*, 2021). The cathode mix (~2 mg) was loaded through a brass funnel (Ø6 mm), the electrolyte, $Na_4(B_{12}H_{12})(B_{10}H_{10})$ (~60 mg), was loaded on top of the cathode mix without prepressing the powder (10 mm). The cathode and electrolyte powder was pressed at 300 MPa for 3 minutes. Na on Cu-foil was placed onto the electrolyte. The stack pressure was set to ~1 MPa.

Electrochemical measurements using TiS_2 as active cathode material were conducted using a cathode composite consisting of TiS_2 (Aldrich), $Na(CB_8H_9)_{0.04}(CB_9H_{10})_{0.96}$ and Carbon Super C65 with a 65:25:10 weight ratio. The composite was prepared by mixing $Na(CB_8H_9)_{0.04}(CB_9H_{10})_{0.96}$ with TiS_2 for 5 minutes using mortar and pestle, then adding the carbon and mixing for another 15 minutes. For measurements in PEEK cells, a brass funnel

(Ø6 mm) was used to place the cathode composite (~6.5 mg) in cells with a diameter of 10.3 mm, subsequently 50 mg $Na(CB_8H_9)_{0.04}(CB_9H_{10})_{0.96}$ was placed in the cell and the cell was pressed at 350 MPa for 3 minutes. For measurements in APTOX cells, ~4.5 mg of cathode composite was placed in a 5 mm pellet pressing die, 25 mg of $Na(CB_8H_9)_{0.04}(CB_9H_{10})_{0.96}$ was placed on top and pressed at 500 MPa for 3 minutes. All cells had ~15 mg CAM per cm^2. Freshly polished Na on Cu-foil was placed opposite to the CAM. The stack pressure was set to ~2 MPa. The APTOX-Pmon cell used to obtain in-house *operando* PXRD data, was assembled using the same method, however ~11 mg of cathode composite was used.

Table S3 Specification of two different demo-systems of the APTOX cell. *The cathode material contains a Cr_2O_3 impurity, the actual mass of $NaCrO_2$ was later determined to be ~65 % noted in parenthesis.

Model	PXRD-facility	Window configuration	Current Collector	Stack Pressure	Temperature (°C)	Cathode active material	Mass of CAM (mg)
APTOX-Pmon	In-house, Ag source (λ = 0.5594 Å)	1 x 0.5 mm and 1 x 2 mm Al-alloy (EN AW-5754)	Copper foil	~2.5 MPa	60	TiS_2	7.1
APTOX-Spring	Synchrotron, DanMAX (MAX IV), (λ = 0.354241 Å)	2 x 2 mm Al-alloy (EN AW-5754)	Copper foil	1 MPa	60	$NaCrO_2$	1.4 (0.9)*

Operando Powder X-Ray Diffraction

The APTOX-Spring cell was implemented at the MAX-IV synchrotron at the DanMAX beamline with λ = 0.354241 Å using the DECTRIS PILATUS3 X 2M CdTe area detector. The acquisition time of each scan was 1 second and then a 40 second latency time. The APTOX-Pmon cell was mounted using a custom-made stage on a STOE STADI P (Ag $K\alpha_1$, λ = 0.5594 Å). The acquisition time was 10 minutes per scan.

Rietveld refinement analysis of the *operando* SR-PXRD data was done based on the structures published by Bo et al. (Bo *et al.*, 2016) (P’3), and Jakobsen et al. (O3, O’3 and O’3-E) (Jakobsen *et al.*, 2022). Rietveld refinements of the TiS_2 system were performed using TiS_2 (*P*-3*m*1)(Chianelli *et al.*, 1975) and $NaCB_9H_{10}$ (*P*31*c*)(Brighi *et al.*, 2020) for all scans adding Na (*Im*-3*m*)(Barrett, 1956) and $Na_{0.55}TiS_2$ (*R*3*m*)(Wiedemann *et al.*, 2019) for scan 13 and 24. Both systems were refined using FullProf Suite.

Chapter 9 Results

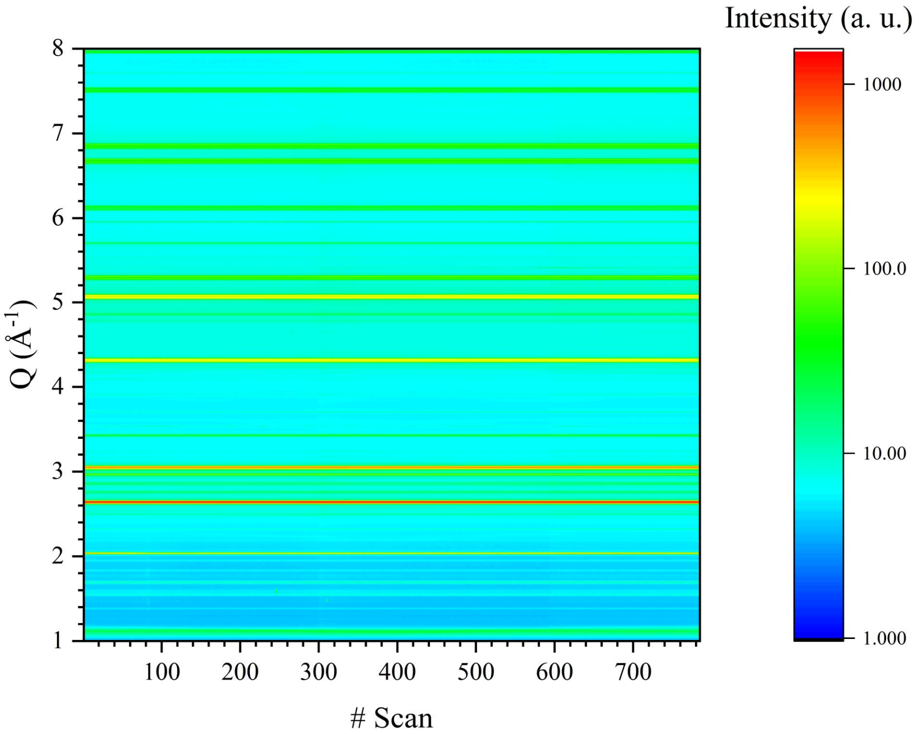

Figure S17 Full contour plot (logarithmic intensity scale) of the *operando* SR-PXRD data collected for Na|$Na_4(B_{12}H_{12})(B_{10}H_{10})$|$NaCrO_2$ at DanMAX using APTOX-Spring.

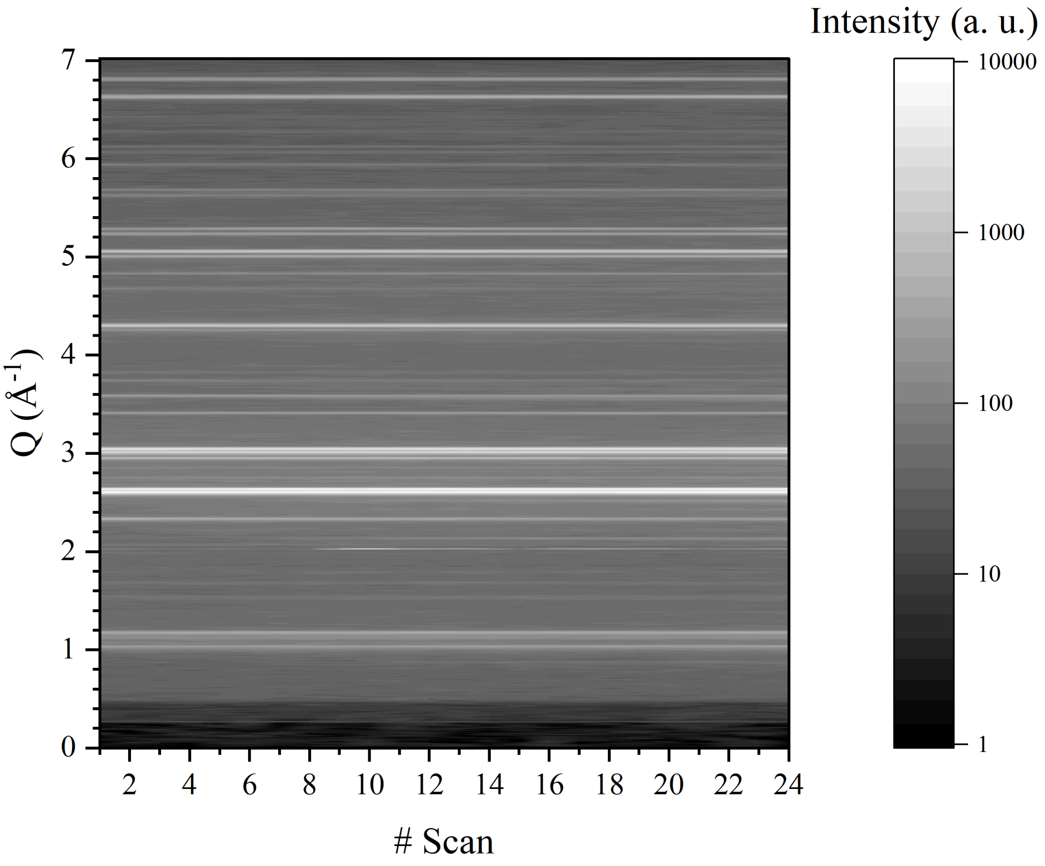

Figure S18 Full contour plot of the collected in-house *operando* PXRD data (logarithmic intensity scale) of the $Na|Na(CB_8H_9)_{0.04}(CB_9H_{10})_{0.96}|TiS_2$ cell measured using APTOX-Pmon.

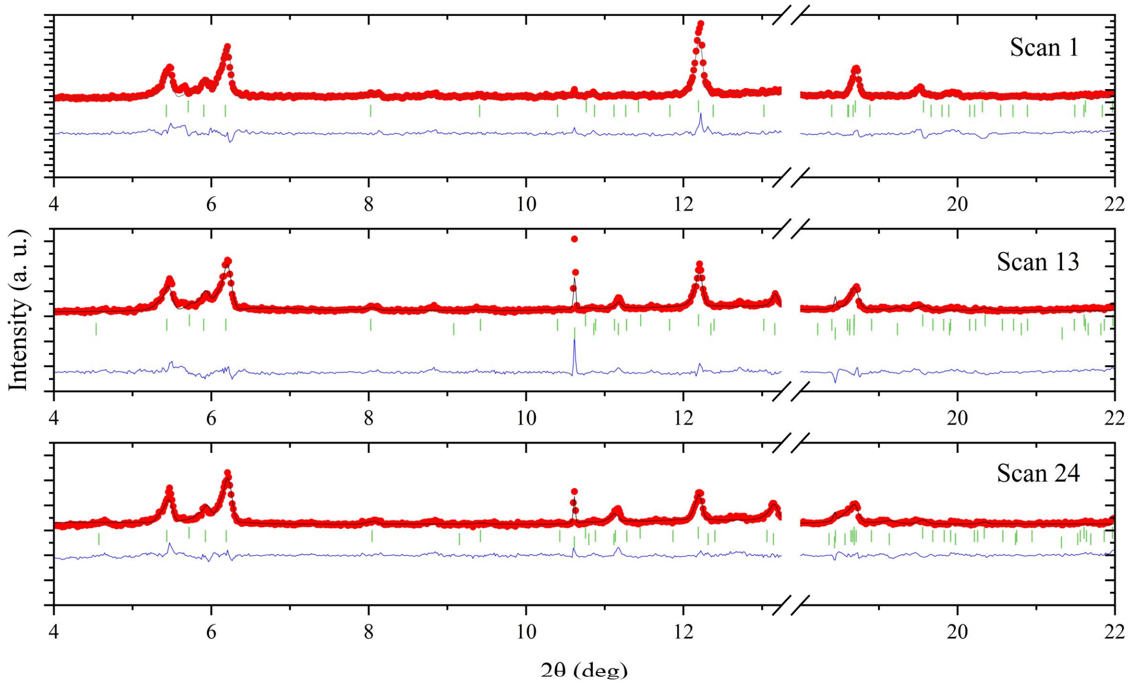

Figure S19 Reitveld refinements scans 1, 13 and 24 of the Na|$Na(CB_8H_9)_{0.04}(CB_9H_{10})_{0.96}$|$TiS_2$ cell measured using APTOX-Pmon. Chi^2 for fits were 2.60, 2.42 and 1.62 for scan 1, 13 and 24, respectively.

References

Asakura, R., Duchêne, L., Payandeh, S., Rentsch, D., Hagemann, H., Battaglia, C. & Remhof, A. (2021). *ACS Appl. Mater. Interfaces* **13**, 55319–55328.

Barrett, C. S. (1956). *Acta Cryst.* **9**.

B. Grinderslev, J., S. Kjær, T. S., N. Skov, L. & R. Jensen, T. (2025). *Journal of Materials Chemistry A* **13**, 15031–15039.

Bo, S.-H., Li, X., Toumar, A. J. & Ceder, G. (2016). *Chem. Mater.* **28**, 1419–1429.

Brighi, M., Murgia, F. & Černý, R. (2020). *Cell Reports Physical Science* 100217.

Chianelli, R. R., Scanlon, J. C. & Thompson, A. H. (1975). *Materials Research Bulletin* **10**, 1379–1382.

Huang, Y., Černý, R., Battaglia, C. & Remhof, A. (2023). *J Mater Sci* **58**, 7398–7406.

Jakobsen, C. L., Brighi, M., Andersen, B. P., Ducrest, G., Černý, R. & Ravnsbæk, D. B. (2022). *Journal of Power Sources* **535**, 231317.

Wiedemann, D., Suard, E. & Lerch, M. (2019). *RSC Adv.* **9**, 27780–27788.

Zheng, L., Bennett, J. C. & Obrovac, M. N. (2019). *J. Electrochem. Soc.* **166**, A2058.
